# Nanoprobes‐Assisted Multichannel NIR‐II Fluorescence Imaging‐Guided Resection and Photothermal Ablation of Lymph Nodes

**DOI:** 10.1002/advs.202003972

**Published:** 2021-02-15

**Authors:** Xiaoxiao Fan, Yirun Li, Zhe Feng, Guoqiao Chen, Jing Zhou, Mubin He, Lan Wu, Shengliang Li, Jun Qian, Hui Lin

**Affiliations:** ^1^ Department of General Surgery Sir Run Run Shaw Hospital School of Medicine Zhejiang University Hangzhou 310000 P. R. China; ^2^ State Key Laboratory of Modern Optical Instrumentations Centre for Optical and Electromagnetic Research College of Optical Science and Engineering International Research Center for Advanced Photonics Zhejiang University Hangzhou 310058 P. R. China; ^3^ College of Pharmaceutical Sciences Soochow University Suzhou 215123 P. R. China; ^4^ Center of Super‐Diamond and Advanced Films (COSDAF) Department of Chemistry City University of Hong Kong 83 Tat Chee Avenue Kowloon Hong Kong 999077 P. R. China

**Keywords:** imaging‐guided, lymphadenectomy, multichannel, nanoprobes, NIR‐II fluorescence imaging, photothermal ablation

## Abstract

Lymph node metastasis is a major metastatic route of cancer and significantly influences the prognosis of cancer patients. Radical lymphadenectomy is crucial for a successful surgery. However, iatrogenic normal organ injury during lymphadenectomy is a troublesome complication. Here, this paper reports a kind of organic nanoprobes (IDSe‐IC2F nanoparticles (NPs)) with excellent second near‐infrared (NIR‐II) fluorescence and photothermal properties. IDSe‐IC2F NPs can effectively label lymph nodes and helped achieve high‐contrast lymphatic imaging. More importantly, by jointly using IDSe‐IC2F nanoparticles and other kinds of nanoparticles with different excitation/emission properties, a multichannel NIR‐II fluorescence imaging modality and imaging‐guided lymphadenectomy is proposed. With the help of this navigation system, the iatrogenic injury can be largely avoided. In addition, NIR‐II fluorescence imaging‐guided photothermal treatment (“hot” strategy) can ablate those metastatic lymph nodes which are difficult to deal with during resection (“cold” strategy). Nanoprobes‐assisted and multichannel NIR‐II fluorescence imaging‐guided “cold” and “hot” treatment strategy provides a general new basis for the future precision surgery.

## Graphical abstract

A novel kind of second near‐infrared (NIR‐II) fluorescent nanoprobe is synthesized to label and trace lymph nodes (LNs). Together with other kinds of NIR‐II fluorescent NPs with different optical features, multichannel fluorescence imaging‐guided surgeries are successfully conducted. Photothermal ablation of metastatic LNs is introduced as a good complement to surgery. This platform offers a novel perspective through which a more precise surgery can be conducted.

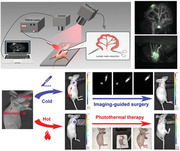

## 1 Introduction

Cancer is one of the leading causes of human death and casts a serious threat on human health.^[^ ^1^ ^]^ Tumor metastasis is directly or indirectly responsible for the majority of cancer deaths.^[^ ^1^ ^]^ Lymph node (LN) metastasis is one of the common routes for cancer dissemination.^[^ ^2^ ^]^ Currently, surgery, including radical resection and organ transplantation, is the only way that can potentially cure the cancer. Radical resection of metastatic LNs along with primary tumor is crucial for a successful surgery.^[^ ^3^ ^]^ Two kinds of LNs are concerned by surgeons. One is sentinel lymph nodes (SLNs) which are identified as the first station when the tumor cells spread via lymphatic system.^[^ ^4^ ^]^ The other type of lymph nodes is the regional lymph nodes (RLN), which are identified as those LNs draining from all areas of the primary tumor site.^[^ ^5^ ^]^ However, how to rapidly and accurately pinpoint SLNs or RLNs under the complexly surgical environment remains a big challenge. In addition, LNs typically distribute along the major blood vessels. The acute injury on major blood vessels often occurs in the process of LNs resecting and leads to a dilemma during abdominal surgery. Besides, iatrogenic ureteral injury is another troublesome complication in radical surgery for colorectal cancer^[^ ^6^ ^]^ and gynecologic cancer.^[^ ^7^ ^]^ Thus, how to avoid the major blood vessels injury and iatrogenic ureteral injury during lymphadenectomy is another issue concerned by surgeons. Notably, sometimes metastatic LNs locate in a very dangerous position (e.g., the metastatic LNs are close to the aortaventralis), which usually induce a failing resection. How to deal with these metastatic LNs under the circumstances is also a difficulty that must frequently be faced by surgeons.

To solve the first above‐mentioned problem in clinical practice, several methods have been developed to trace the LNs. Among them, two kinds of tracers are most widely used, which are radiotracers (e.g., Tc99m‐labeled colloids) and chromophores (e.g., methylene blue, nanocarbon, etc.), respectively.^[^ ^4^ ^]^ These tracers help to detect deep LNs and find LNs in the surgical area when the tissue is cut open, respectively. However, the flaws of these tracers are obvious. The radiation from radiotracers is harmful to human's health, while the visible chromophores cannot be visualized below the surface of tissue.^[^ ^4^ ^]^ Fluorescence imaging‐guided surgery has shown great advantages in differentiating normal and pathologic structures during surgical procedures.^[^ ^8^ ^]^ Because of the lower autofluorescence and better tissue penetration, fluorophores assisted the near‐infrared (NIR) fluorescence imaging generally arouse particular interests to surgeons and researchers.^[^ ^8^ ^]^ Currently, the most commonly used agent for NIR fluorescence imaging in clinical practice is indocyanine green (ICG), and its emission signal is acquired in the first near‐infrared window (NIR‐I, 760–900 nm).^[^ ^9^ ^]^ With the development of the optical imaging technology and probes, fluorescence imaging in the second near‐infrared window (NIR‐II, 900–1700 nm) displays great potential in clinical practice.^[^ ^10^ ^]^ Compared to traditional visible and NIR‐I fluorescence imaging, NIR‐II fluorescence imaging presents obvious advantages, such as improvements in spatial resolution, penetration depth, and signal‐to‐background ratio (SBR).^[^ ^11^ ^]^ So far, many kinds of NIR‐II fluorophores, such as single‐walled carbon nanotubes,^[^ ^12^ ^]^ quantum dots,^[^ ^13^ ^]^ rare‐earth doped nanoparticles,^[^ ^14^ ^]^ organic small molecules,^[^ ^11b^ ^]^ and organic nanoparticles,^[^ ^10^ ^]^ have been developed. Among all of them, organic fluorophores encapsulated nanoparticles are regarded as ideal materials for in vivo imaging because of the excellent biocompatibility and processability.^[^ ^15^ ^]^ Moreover, the existence of various kinds of NIR‐II fluorophores with different excitation/emission properties make it possible to establish a multichannel NIR‐II fluorescence imaging modality, which is very useful for surgical navigation.

In this study, we developed a kind of organic NIR‐II fluorophore IDSe‐IC2F and its nanoparticles with bright NIR‐II fluorescence for lymphatic system imaging‐guided resection and photothermal ablation. Thanks to the high spatial resolution of NIR‐II fluorescence imaging modality, the LNs of rats stained with IDSe‐IC2F NPs presented as a “ring” enhancement. Furthermore, a dual‐channel NIR‐II fluorescence imaging platform for lymphadenectomy was constructed based on IDSe‐IC2F NPs and another kind of NIR‐II fluorescent NPs, and this imaging navigation system can help to avoid the severe complications during the lymphadenectomy in a rat model. We also demonstrated triple‐channel NIR‐II fluorescence imaging by using IDSe‐IC2F NPs and two other kinds of NPs, showing LNs, vessels, and ureters at the same time simultaneously. Finally, two treatment strategies, namely, “cold” strategy and “hot” strategy, respectively, were proposed to deal with potentially metastatic LNs. “Cold” strategy means NIR‐II fluorescence imaging‐guided resection of metastatic LN, whereas “hot” strategy stands for NIR‐II image‐guided photothermal ablation. This novel navigation platform provides a promising and powerful solution to the precision surgery.

## 2 Results

### 2.1 Characterization of IDSe‐IC2F NPs

**Figure 1:**
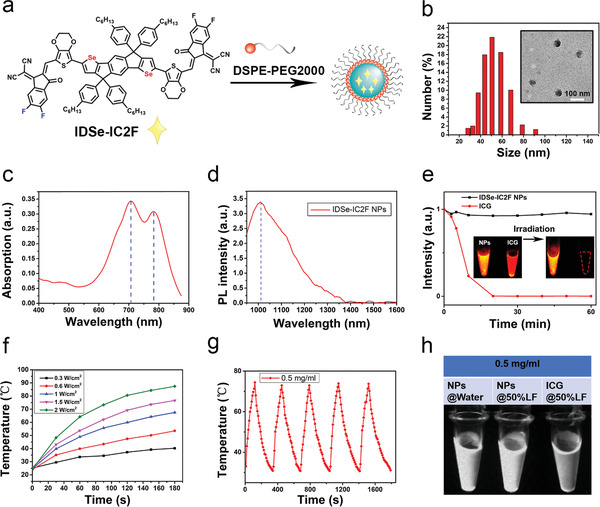
Characterizations of IDSe‐IC2F NPs. a) Schematic illustration of the fabrication process. b) A representative TEM image and DLS result of IDSe‐IC2F NPs. Scale bar: 100 nm. c) Absorption and d) photoluminescence (PL) spectra of IDSe‐IC2F NPs in water; e) photostability test for IDSe‐IC2F NPs (0.1 mg mL^−1^) and ICG (0.1 mg mL^−1^) in water. Aqueous dispersion was upon continuous irradiation for 60 min (power density: 1.5 W cm^−2^); f) temperature changes of aqueous dispersion of DSe‐IC2F NPs after irradiation of 793 nm laser with different power densities; g) temperature change curves of aqueous dispersion of IDSe‐IC2F NPs with the irradiation of 793 nm laser on and off (1.5 W cm^−2^, five cycles); h) NIR‐II fluorescence image of IDSe‐IC2F NPs (0.5 mg mL^−1^) in water and in lymphatic fluid (LF) and ICG (0.5 mg mL^−1^) in LF (optical filter: 1000 nm LP, power density: 7.5 mW cm^−2^).

The IDSe‐IC2F was designed and synthesized described in our previous work. To received good NIR‐II emission, the strong donor 7,7′‐(4,4,9,9‐tetrakis(4‐hexylphenyl)‐4,9‐dihydro‐s‐indaceno[1,2‐*b*:5,6‐*b*′]bis(selenophene)‐2,7‐diyl)bis(2,3‐dihydrothieno[3,4‐*b*][1,4]dioxine‐5‐carbaldehyde) was conjugated with 2‐(5,6‐difluoro‐3‐oxo‐2,3‐dihydro‐1H‐inden‐1‐ylidene)malononitrile to form a A‐*π*‐D‐*π*‐A backbone, with a *π*‐bridge of ethylene dioxothiophene (EDOT) unit. To improve the water solubility, IDSe‐IC2F molecules were encapsulated into NPs by using amphiphilic polymer DSPE‐PEG_2000_ (**Figure** **1**a). The transmission electron microscopy (TEM) revealed IDSe‐IC2F NPs had a uniform sphere structure with a mean diameter of ≈52 nm, which consisted with the results of dynamic light scattering (DLS) measurement (Figure 1b). In addition, the mean diameters of IDSe‐IC2F NPs in water and phosphate buffered saline (PBS) (1×) were similar (≈58 nm, Figure S1, Supporting Information). The zeta potential of NPs in water was 0.146 mV (Figure S2, Supporting Information). Two absorption peaks of IDSe‐IC2F NPs were observed, which located at ≈710 and ≈790 nm, respectively (Figure 1c). The molar extinction coefficient at 793 nm wavelength was calculated as 78.4 mm ^−1^ cm^−1^. The photoluminescence (PL) peak was detected at ≈1010 nm (Figure 1d), and a fluorescence tail could extend to 1300 nm. The NIR‐II fluorescence lifetime of IDSe‐IC2F NPs in a glass capillary tube was measured as 281 ps by using our home‐built NIR‐II fluorescence lifetime confocal microscopic imaging system^[^ ^16^ ^]^ (Figure S4, Supporting Information). Compared to ICG (0.1 mg mL^−1^), the NPs (0.1 mg mL^−1^) displayed higher photostability in water (Figure 1e). The fluorescence intensity loss of IDSe‐IC2F NPs was negligible upon continuous 793 nm laser irradiation for 1 h with a power density of 1.5 W cm^−2^, whereas the fluorescence of ICG was quenched after irradiation (Figure 1e). To further assess the photothermal effect of IDSe‐IC2F NPs, the aqueous dispersion was exposed to a 793 nm laser with different power densities. The temperature could rapidly increase after irradiation (Figure 1f). The photothermal conversion efficiency of NPs was calculated as 38.6% according to the previous method^[^ ^17^ ^]^ and was comparable with previously reported materials.^[^ ^15^, ^18^ ^]^ To evaluate the photothermal stability, IDSe‐IC2F NPs in water were exposed to the laser irradiation with a power density of 1.5 W cm^−2^. The temperature was recorded during the cycles of heating and cooling processes. As presented in Figure 1g, the highest temperature could reach ≈74 °C, and the deterioration of heating behavior of the NPs was negligible after five heating/cooling cycles.

ICG is a widely used fluorophore in clinical practice to trace the LNs. In water, the fluorescence intensity of IDSe‐IC2F NPs (0.1 mg mL^−1^) is higher than that of ICG (0.1 mg mL^−1^) (Figure 1e). We also collected lymphatic fluid (LF) from a thyroid cancer patient who suffered from lymphatic leak after radical thyroidectomy to assess the PL intensity of IDSe‐IC2F NPs and ICG in LF (Figure 1h and Figure S5a, Supporting Information). Different from ICG, the PL intensity of IDSe‐IC2F NPs would not be affected by LF (Figure S5b, Supporting Information). At the same concentration in LF, the PL of IDSe‐IC2F NPs was brighter than that of ICG (Figure 1g).

### 2.2 NIR‐II Fluorescence Imaging of LNs in Mouse Model

**Figure 2:**
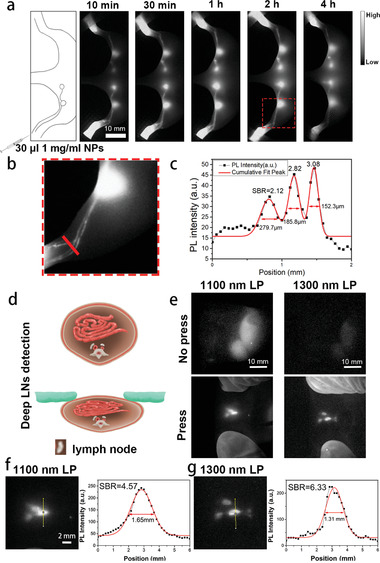
IDSe‐IC2F NPs enable high contrast LN imaging. a) Schematic illustration of injection method and the lymphatic system structure in the hindlimb of mouse, and representative NIR‐II fluorescence images of sciatic and popliteal LNs at different time points after injection of NPs. Scale bar: 10 mm. b) A local magnified NIR‐II fluorescence image of lymph vessels from the injection site to popliteal LN. Scale bar: 10 mm. c) A cross‐sectional fluorescence intensity profile (black) and the Gaussian fit (red) along the red line in (b). d) Schematic illustration of the position change of retroperitoneal LNs in a nude mouse when its abdominal wall was pressed. e) NIR‐II fluorescence images of retroperitoneal LNs of the nude mouse when its abdominal wall was pressed and not pressed. 1100 nm LP (43 mW cm^−2^, 100 ms) and 1300 nm LP (100 mW cm^−2^, 250 ms) optical filters were used, respectively. f,g) The local magnified NIR‐II fluorescence images of retroperitoneal LNs at press status by using the 1100 and 1300 nm LP filter, respectively. Cross‐sectional fluorescence intensity profiles and Gaussian fits along the yellow lines in (f) and (g).

To investigate whether the IDSe‐IC2F NPs could efficiently label the LNs and lymphatic vessels, we first injected the IDSe‐IC2F NPs into the rear paw of the nude mouse. Both sciatic and popliteal lymph nodes could be clearly detected based on NIR‐II fluorescence imaging beyond 1100 nm after injection (43 mW cm^−2^, **Figure** **2**a). The NIR‐II PL intensity gradually reached to the maxima and maintained at least for 4 h (Figure 2a). Furthermore, three main lymph vessels could be detected draining from the primary injection site to the popliteal LN (Figure 2b), which mainly benefitted from the high spatial resolution and penetration depth of NIR‐II fluorescence imaging. A cross‐sectional fluorescence intensity profile showed the full width at half‐maximum from 152.3 to 279.7 µm (Figure 2c). The maximum SBR of lymph vessels could reach 3.08.

As previous studies reported, the thickness between the surface of skin and the retroperitoneal LNs in mouse was ≈1.5 cm.^[^ ^19^ ^]^ When the abdominal wall was pressed, the depth reduced to ≈0.5 cm.^[^ ^19^ ^]^ Interestingly, ≈30 min after injecting the IDSe‐IC2F NPs into the rear paw, the retroperitoneal LNs could be easily and clearly observed (Figure 2e) when the mouse's abdominal wall was pressed (Figure 2d). In addition, compared to the results obtained from the NIR‐II fluorescence imaging beyond 1100 nm, sharper images were acquired when conducting NIR‐II fluorescence imaging beyond 1300 nm. As shown in Figure 2f,g, the SBR (6.33) of NIR‐II fluorescence image beyond 1300 nm was higher than that beyond 1100 nm (4.57).

Furthermore, we compared the difference between ICG (the most widely used fluorophore to trace the lymph nodes in the clinical practice) and our NPs after a local administration in vivo. Strong NIR‐II fluorescence signals of ICG from femoral veins and liver were observed 1 h after the injection (Figure S6, Supporting Information). It is worth mentioning that as a small molecule, ICG leaked into the blood circulation system after local administration much more quickly and easily, when compared to our NPs. We also performed a 10 d tracing to analyze the distribution of IDSe‐IC2F NPs after a local administration in vivo. The NIR‐II fluorescence signals of LN were persistent. Besides in the injection site and LN, the signals of NIR‐II fluorescence in other organs were negligible (Figure S7, Supporting Information).

### 2.3 Labeling Different LNs of Rat Using IDSe‐IC2F NPs

**Figure 3:**
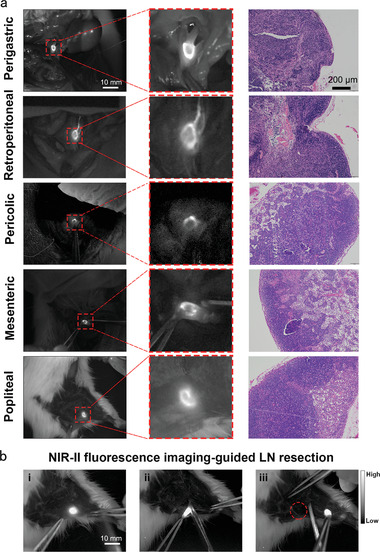
NIR‐II fluorescence images of different kinds of LNs in rats labeled with IDSe‐IC2F NPs. a) Original NIR‐II fluorescence images, local magnified NIR‐II fluorescence images and the H&E staining microscopic images of perigastric LN, retroperitoneal LN, pericolic LN, mesenteric LN and popliteal LN. Scale bar of NIR‐II fluorescence images: 10 mm; scale bar of H&E staining microscopic images: 100 µm. b) NIR‐II fluorescence imaging‐guided lymphadenectomy of popliteal LN in a rat.

Encouraged by the above results in nude mouse, the experiment was further carried out to label different LNs of rats. We studied five kinds of LNs from different areas, which represented the five kinds of most common metastatic LNs in cancer patients. There is a LN permanently locating at the lesser curvature of stomach, which collected the lymph fluid from the stomach wall and was very close to the left gastric vessels in rat. However, this LN is difficult to be identified in the rat's abdominal cavity in incandescent lighting. We injected IDSe‐IC2F NPs into the gastric wall and used an incandescent lamp with NIR‐II emission tail to provide the background. Strong fluorescence signal in NIR‐II window could be observed about 10 min after injection (**Figure** **3**a and Figure S8, Supporting Information). For retroperitoneal LN, we observed it by injecting the IDSe‐IC2F NPs into the rat's foot pad. 20 min after injection, bright NIR‐II fluorescence signal was detected in retroperitoneal LN. The pericolic LN could be detected by injecting IDSe‐IC2F NPs into the wall of colon. There are several LNs permanently locating at the root of mesenteries and collected the lymphatic fluid from the small intestine in rat.^[^ ^20^ ^]^ Based on this anatomical structure, we labeled and imaged the mesenteric LNs by injecting the NPs into follicles. More interestingly, we recorded the entire process of lymphatic drainage from the follicles to the LNs (Video S1, Supporting Information). At last, we used IDSe‐IC2F NPs to trace the popliteal LN of rat. Unexpectedly, assisted by IDSe‐IC2F NPs, the fluorescence imaging characteristic of LNs in NIR‐II window was “ring” enhancement (Figure 3a). Low scattering of NIR‐II fluorescence signal helps us to detect more detailed structure of LNs. All the LNs were confirmed by the hematoxylin and eosin (H&E) staining. Furthermore, we performed NIR‐II fluorescence imaging‐guided resection of popliteal LN (Figure 3b). Under the navigation of imaging, the popliteal LN was precisely resected without any LN tissues remained.

### 2.4 Observing LNs and Vessels Simultaneously by Dual‐Channel NIR‐II Fluorescence Imaging

**Figure 4:**
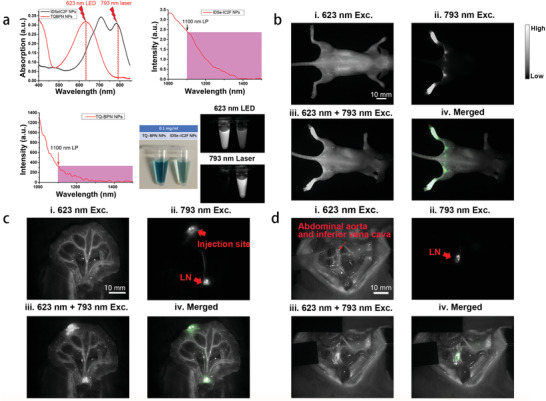
Dual‐channel NIR‐II fluorescence images of LNs and blood vessels. a) The differences of absorption and photoluminescence spectra between IDSe‐IC2F NPs and TQ‐BPN NPs, and the NIR‐II fluorescence images of IDSe‐IC2F NPs and TQ‐BPN NPs under the irradiation of 793 nm laser and 623 nm LED, respectively. b) i) Whole‐body blood vessel imaging of a mouse intravenously injected with TQ‐BPN NPs, under the excitation of 623 nm LED (before injection of IDSe‐IC2F NPs, 30 mW cm^−2^, exposure time (HG): 100 ms, 1100 nm LP); ii) sciatic and popliteal LNs imaging of the mouse treated with IDSe‐IC2F NPs, under the excitation of 793 nm laser (40 mW cm^−2^, HG: 100 ms, 1100 nm LP); iii) simultaneous imaging of whole‐body blood vessels and LNs, under the excitation of both 623 nm LED and 793 nm laser; iv) merged image of (ii) and (iii). Scale bar: 10 mm. c) i) Mesenteric vessels imaging of a rat receiving caudal vein injection of TQ‐BPN NPs, upon the excitation of 623 nm LED (before injection of IDSe‐IC2F NPs); ii) LNs imaging of the rat, which was injected with IDSe‐IC2F NPs from follicles and excited with the 793 nm laser; iii) simultaneous imaging of mesenteric vessels and LN, under the excitation of both 623 nm LED and 793 nm laser; iv) merged image of (ii) and (iii). Scale bar 10 mm; d) i) imaging of major blood vessels in retroperitoneal area of a rat intravenously injected with TQ‐BPN NPs (before injection of IDSe‐IC2F NPs, 623 nm LED excitation); ii) retroperitoneal LNs imaging of the rat treated with IDSe‐IC2F NPs (793 nm laser excitation); iii) simultaneous imaging of major blood vessels and LNs, under the excitation of both 623 nm LED and 793 nm laser; iv) merged image of (ii) and (iii). Scale bar: 10 mm.

In our previous study, we reported a kind of bright aggregation‐induced emission nanoprobes called TQ‐BPN NPs and its application for in vivo functional fluorescence bioimaging in NIR‐II region.^[^ ^21^ ^]^ The absorption peak of the TQ‐BPN NPs was at ≈630 nm, and the emission peak was at ≈810 nm (**Figure** **4**a). More importantly, the quantum yield (QY) of TQ‐BPN NPs in NIR‐II region reached 2.8%, and an intense emission tail of TQ‐BPN NPs beyond 1100 nm was observed (Figure 4a). An unambiguous image showing the whole‐body blood vessels of mouse and mesenteric vessels of rat could be acquired by using TQ‐BPN NPs‐based NIR‐II fluorescence imaging under 623 nm LED excitation (Figure S9, Supporting Information). Furthermore, different from our newly synthesized IDSe‐IC2F NPs, TQ‐BPN NPs were barely excited by the 793 nm laser (Figure 4a). The excitation characteristic difference between the two kinds of NPs is the basis for achieving dual‐channel NIR‐II fluorescence bioimaging.

We established a home‐made dual‐channel NIR‐II fluorescence imaging system which mainly contained an InGaAs camera, a 793 nm CW laser, and a 623 nm LED (Figure S10, Supporting Information). Because of the wide emission spectrum of the 623 nm LED, it not only excited the TQ‐BPN NPs but also provided the visual background of surgical area. To present the advantage of dual‐channel NIR‐II fluorescence imaging, we first performed whole‐body blood vessels imaging (623 nm LED excitation (Exc.), Figure 4b‐i of a nude mouse intravenously injected with TQ‐BPN NPs (200 µL, 1 mg mL^−1^). Second, we injected the IDSe‐IC2F NPs into foot pad to acquire the images of popliteal and sciatic LNs (793 nm laser Exc., Figure 4b‐ii). As TQ‐BPN NPs were hardly excited by 793 nm laser, a high‐SBR image contained LNs alone was acquired (Figure 4b‐ii). When the mouse was excited by both the 623 nm LED (30 mW cm^−2^) and 793 nm laser (10 mW cm^−2^), an image containing both blood vessels and LNs was achieved (Figure 4b‐iii). We further coded the pseudo‐color (green) of the LNs image (Figure 4b‐ii) by software (Adobe Photoshop) and accurately merged the transformed figure with Figure 4b‐iii (Figure 4b‐iv).

To simulate the surgical environment, we performed the dual‐channel NIR‐II fluorescence imaging in the abdominal cavity of a rat. First, we intravenously injected the TQ‐BPN NPs into rat. An image with various mesenteric vessels was acquired (Figure 4c‐i). Next, we acquired the image of mesenteric LNs by injecting the IDSe‐IC2F NPs into rat's aggregated lymphatic follicle (Figure 4c‐ii). After obtaining an image with both blood vessels and LNs under both 623 nm LED and 793 nm laser excitation (Figure 4c‐iii), we overlapped Figure 4c‐ii with Figure 4c‐iii. The green pseudo‐color could help us easily discriminate the mesenteric LNs from the surrounding rich mesenteric vessels. The similar method was also conducted to synchronously visualize the LNs and major blood vessels in the retroperitoneal area (Figure 4d).

### 2.5 Dual‐Channel NIR‐II Fluorescence Imaging‐Guided LNs Resection

**Figure 5:**
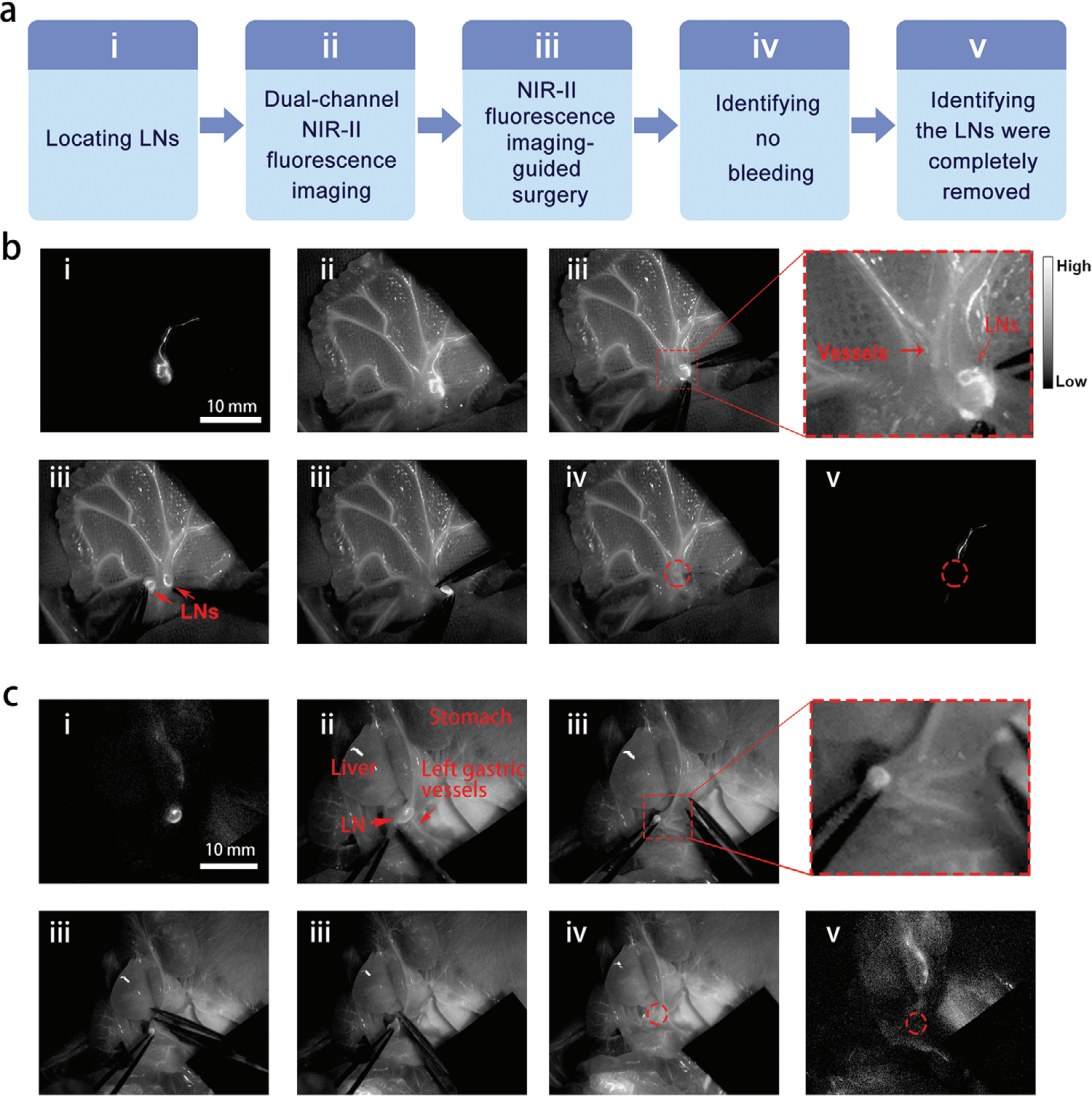
Dual‐channel NIR‐II fluorescence imaging‐guided lymphadenectomy on rats. a) Brief description of the process. b) i) The precisely located mesenteric LNs labeled with IDSe‐IC2F NPs under 793 nm laser excitation; ii) dual‐channel fluorescence imaging of LNs and blood vessels (labeled with TQ‐BPN NPs) under the excitation of both 793 nm laser and 623 nm LED; iii) the process of resection (two LNs were detected and removed); iv) no obvious bleeding was observed; v) no residual LN was detected. Scale bar: 10 mm. c) i) The located gastric LNs labeled with IDSe‐IC2F NPs under 793 nm laser excitation; ii) dual‐channel fluorescence imaging of LNs and gastric vessels (labeled with TQ‐BPN NPs) under the excitation of both 793 nm laser and 623 nm LED; iii) the process of resection (the LN was very close to the left gastric artery); iv) no obvious bleeding was observed; v) no residual LN was detected. Scale bar: 10 mm. Red circles: the position of LNs (after resection).

To assess the application value of dual‐channel NIR‐II fluorescence imaging, we further performed the LNs resection guided by this imaging system. The main steps were demonstrated in **Figure** **5**a, which could be briefly described as following: identifying the location of LNs, dual‐channel NIR‐II fluorescence imaging‐guided resection, confirming there were no active bleedings, and identifying there were no residual LNs. TQ‐BPN NPs and IDSe‐IC2F NPs were injected into the tail vein and small intestine follicle, respectively. Under the 793 nm laser irradiation, we could accurately and rapidly find the location of mesenteric LNs and the lymphatic vessels (Figure 5b‐i). Then, we turned on the 623 nm LED to detect the major blood vessels (Figure 5b‐ii). Depending on the dual‐channel NIR‐II fluorescence imaging, we could carefully separate the LNs from the blood vessels and completely resect them (Figure 5b‐iii and Video S2, Supporting Information). Interestingly, two LNs were labeled, and both were resected completely. Under the 623 nm LED excitation, we could easily observe whether there was the existence of active bleeding after surgery (Figure 5a‐iv). Moreover, we could also detect whether there were residual LNs in the surgical area under the irradiation of 793 nm (Figure 5a‐v).

The similar modality was also used to remove the gastric LNs (Figure 5c and Video S3, Supporting Information). As a control, we also performed a single‐channel NIR‐II fluorescence imaging‐guided gastric lymphadenectomy. During this surgery, the blood vessels could not be detected. Because the LN closely located to the left gastric blood vessels, major bleeding happened during the resection (Video S4, Supporting Information).

### 2.6 Dual‐Channel NIR‐II Fluorescence Imaging (LNs and Ureters) and Imaging‐Guided Lymphadenectomy

**Figure 6:**
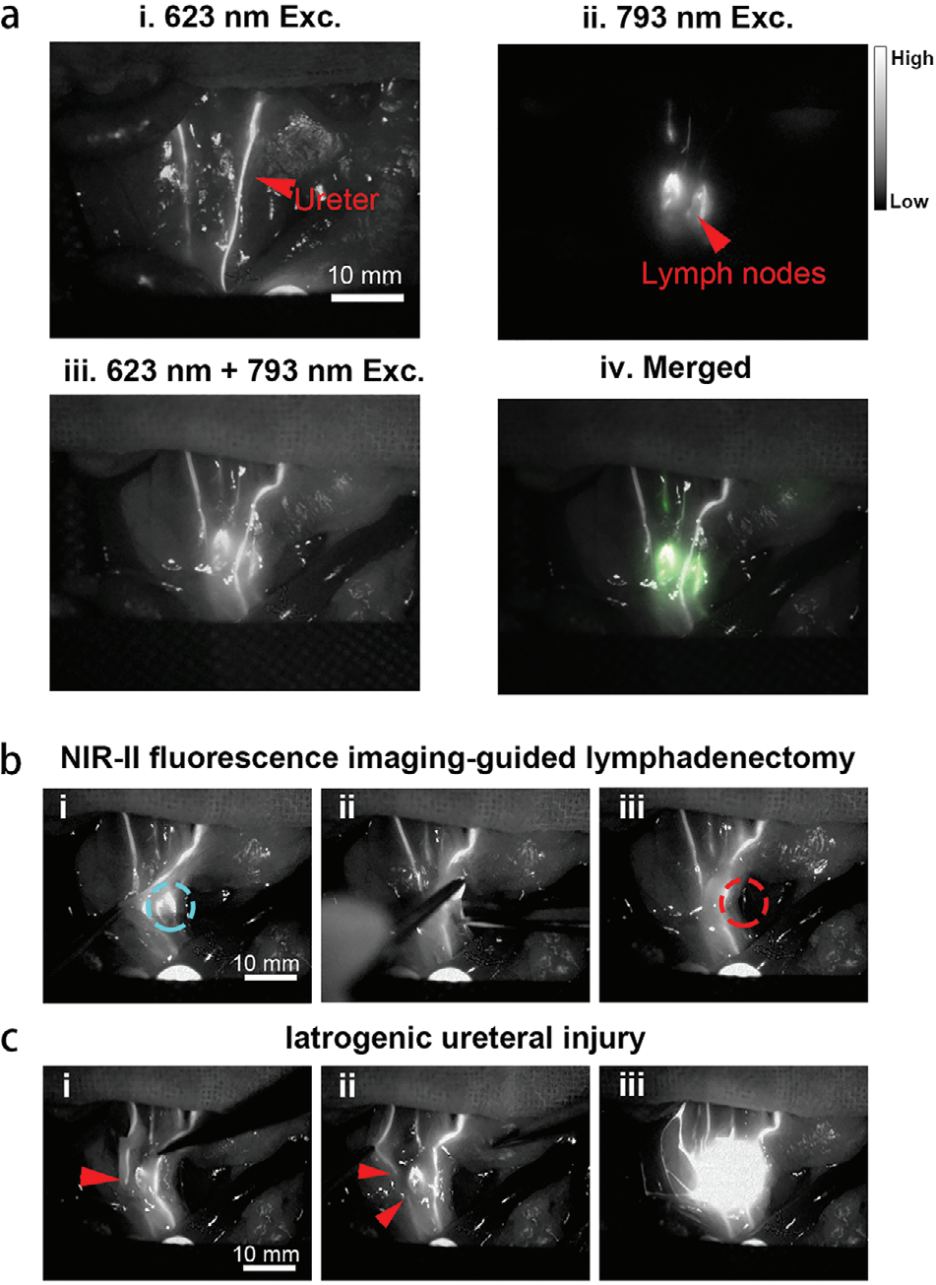
Dual‐channel NIR‐II fluorescence imaging of retroperitoneal LNs and ureters and imaging‐guided lymphadenectomy on rats. a) i) Retrograde ureteropyelography via TQ‐BPN NPs based NIR‐II fluorescence imaging (623 nm LED excitation, before the injection of IDSe‐IC2F NPs); ii) NIR‐II fluorescence image of retroperitoneal LNs labeled with IDSe‐IC2F NPs, under the excitation of 793 nm laser; iii) simultaneous imaging of ureters and LNs under the excitation of both 623 nm LED and 793 nm laser; iv) merged image of (ii) and (iii). Scale bar: 10 mm. b) The process of imaging‐guided lymphadenectomy (blue circle: the position of LNs, before resection): i) pulling the ureters aside; ii) resecting the LN; iii) confirming the LN was completely removed (red circle: the position of LNs, after resection). c) Simulative iatrogenic ureteral injury: i,ii) the broken end of ureter could be detected, red arrow: the broken end of ureter; iii) TQ‐BPN NPs leaked into abdominal cavity from the broken end of ureter as revealed by the fluorescence signal.

Similar to clinical practice, we performed retrograde ureteropyelography to detect ureters. We first injected 0.8 mL TQ‐BPN NPs (0.1 mg mL^−1^) into the rat's bladder and acquired the NIR‐II fluorescence images (1100 nm LP) of ureters under the irradiation of 623 nm LED. The ureters could be unambiguously visualized (**Figure** **6**a‐i). Then, we labeled the LNs of rat by injecting IDSe‐IC2F NPs into its right and left foot pads. Under the 793 nm laser irradiation (40 mW cm^−2^), the retroperitoneal LNs could be clearly detected 15 min after injection (Figure 6a‐ii). Under the simultaneous irradiation of 623 nm LED (30 mW cm^−2^) and 793 nm laser (10 mW cm^−2^), we obtained an image showing both the LNs and ureters of rat (Figure 6a‐iii). Figure 6a‐ii was then precisely overlapped on Figure 6a‐iii to achieve a merged image (Figure 6a‐iv).

In the clinical practice, the iatrogenic ureteral injury is a troublesome complication during the retroperitoneal LNs resection. Considering this, we further performed dual‐channel NIR‐II fluorescence imaging guided lymphadenectomy in the area where the LNs located behind the ureters. We successfully resected the LN without injuring the ureters (Figure 6b). First, we determined the location of LN using 793 nm laser excitation alone. Next, 623 nm LED was used to excite the fluorescence signal from ureters, which were labeled with TQ‐BPN NPs. Under the irradiation of 793 nm laser (10 mW cm^−2^) and 623 nm LED (30 mW cm^−2^), we could trace both the LN and ureters simultaneously. After carefully opening the posterior peritoneum, we pulled the ureter aside and exposed the LN. Then, the LN was precisely and completely removed (Video S5, Supporting Information). Moreover, even if the ureteral injury occurred, the dual‐channel NIR‐II fluorescence imaging‐guided surgery could also help surgeons to detect and locate the injury immediately. We simulated the iatrogenic ureteral injury in the rat model. When the ureteral injury happened, the disconnection position of fluorescence signal was observed (Figure 6c‐i,ii) and fluorescent TQ‐BPN NPs leaked into the surgical region (Figure 6c‐iiia and Video S6, Supporting Information).

### 2.7 Triple‐Channel NIR‐II Fluorescence Imaging (LNs, Blood Vessels, and Ureters)

**Figure 7:**
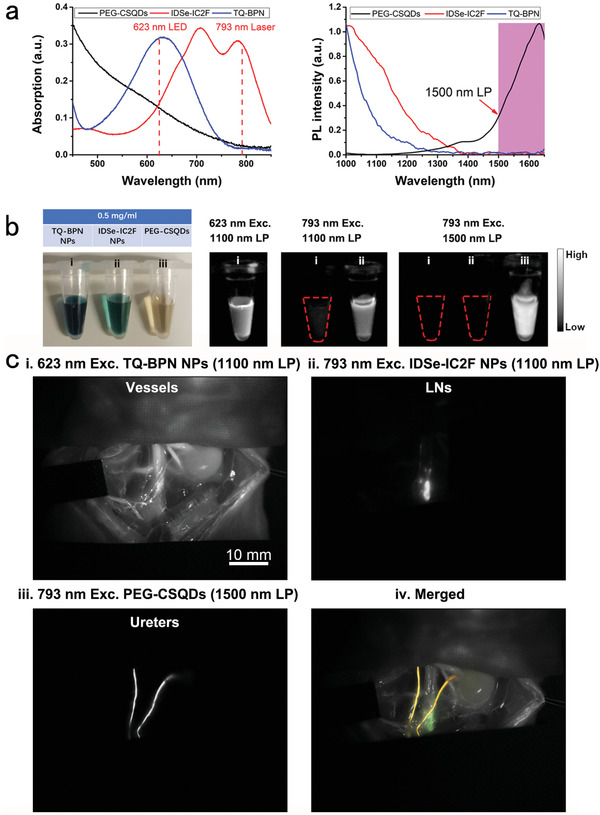
Triple‐channel NIR‐II fluorescence imaging of LNs, blood vessels, and ureters on rats. a) The differences of absorption and PL spectra among the three kinds of NPs (IDSe‐IC2F NPs, TQ‐BPN NPs, and PEG‐CSQDs). b) NIR‐II fluorescence images of these kinds of NPs by using different excitation light sources (623 nm LED and 793 nm laser) and different optical filters (1000 and 1500 nm LP). c) i) NIR‐II fluorescence imaging of major blood vessels labeled with TQ‐BPN NPs (before injection of IDSe‐IC2F NPs, 623 nm LED excitation, and 1100 nm LP); ii) LNs were labeled with IDSe‐IC2F NPs and imaged (793 nm laser excitation, 1100 nm LP); iii) retrograde ureteropyelography via PEG‐CSQDs based NIR‐II fluorescence imaging (793 nm laser excitation, 1500 nm LP); iv) merged image of (i)–(iii).

Another notable NIR‐II fluorescent nanoprobes called PEG‐CSQDs (PEGlyted PbS/CdS quantum dots) was reported by Zhang et al.^[^ ^22^ ^]^ PEG‐CSQDs could be excited by both 793 nm laser and 623 nm LED and emitted bright NIR‐II fluorescent signal with the spectrum peak around 1600 nm (**Figure** **7**a).^[^ ^22^ ^]^ As for IDSe‐IC2F NPs and TQ‐BPN NPs, we barely detected fluorescence in NIR‐IIb (1500–1700 nm) window (Figure 7a). Based on the combination of these three kinds of NPs, we were able to achieve triple‐channel NIR‐II fluorescence imaging by controlling different excitation sources and emission filters (Figure 7b).

The environment of posterior peritoneum area is rather complex. Ureters, LNs, nerves, and various types of blood vessels were distributed in this area (Figure S11, Supporting Information). The operations in posterior peritoneum area are big challenges for surgeons. Therefore, posterior peritoneum area is a deal application scenario for triple‐channel NIR‐II fluorescence imaging. By intravenously injecting TQ‐BPN NPs, an image showing bright blood vessels of rat was acquired under 623 nm LED excitation (Figure 7c‐i). We then visualized LNs as previously described under 793 nm laser irradiation (Figure 7c‐ii). Furthermore, retrograde ureteropyelography was performed by using PEG‐CSQDs as the fluorophore. We excited the rat by the 793 nm laser and recorded the fluorescence signal beyond 1500 nm. A sharp image revealing two ureters was acquired (Figure 7c‐iii). At last, we merged the images in the aforementioned three channels and achieved an image with green LNs and yellow ureters just as the atlas of anatomy (Figure 7c‐iva and Figure S12, Supporting Information).

### 2.8 NIR‐II Fluorescence Imaging‐Guided Resection and Photothermal Ablation of Metastatic LNs

**Figure 8:**
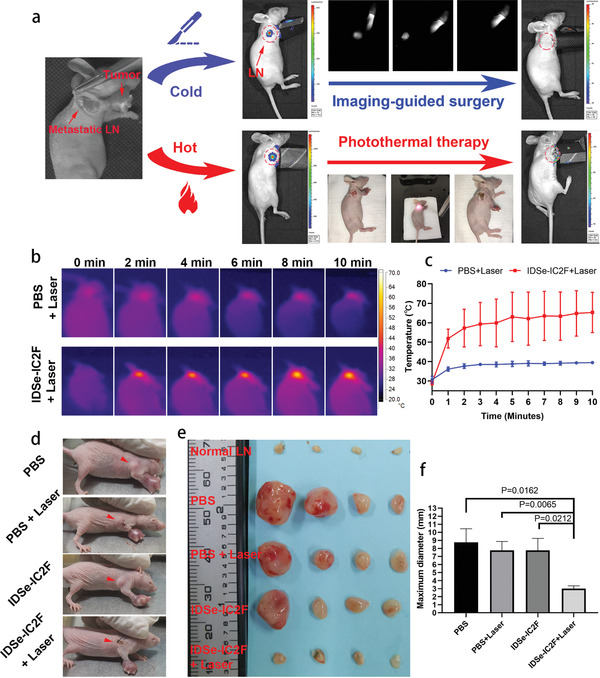
Photothermal ablation efficacy of IDSe‐IC2F NPs on LN metastasis. a) Illustration of the “cold” (resection) and “hot” (photothermal ablation) treatment strategies of LN metastasis. Luciferase signal at the axillary LN could be detected 10 d after injecting 4T1‐luciferase and disappeared after treatment; b) representative photothermal images and c) corresponding temperature changes of metastatic LN in lymphatic metastasis model upon 793 nm laser irradiation (1.5 W cm^−2^). d) Representative digital photographs of tumor bearing mice with various treatments, and the red arrows indicate the metastatic LN. e) The photographs of dissected LNs from the different groups of tumors bearing mice, 10 d post the treatments. f) The maximum diameters of dissected LNs from different groups of mice. Results were presented as mean ± SD, *n* = 4.

To assess the application value of NIR‐II fluorescence imaging and photothermal effect of IDSe‐IC2F NPs in treating metastatic LNs, we first established a luciferase‐transfected 4T1 cell line (4T1‐luciferase) and further constructed a model by injecting the 4T1‐luciferase into the foreleg foot pad of nude mice (Figure S13, Supporting Information). After 10 d, we could detect the swollen axillary LN in the mouse model and a dramatic increase of luciferase signal at the axillary LN by using bioluminescent IVIS imaging system. Two treatment strategies, which were identified as “cold” strategy and “hot” strategy, could be conducted to deal with the metastatic LNs (**Figure** **8**). “Hot” strategy mainly deals with the LNs which located at dangerous positions and is a complement to the “cold” strategy. As for “cold” strategy, we injected IDSe‐IC2F NPs into the paracancerous area. NIR‐II fluorescence imaging was conducted, and strong fluorescence signal could be detected in the swollen axillary LN within 10 min after paracancerous injection. We then performed the NIR‐II fluorescence imaging‐guided resection of LN (Figure 8a and Video S7, Supporting Information). After the operation, the bioluminescent signal of lymph node metastasis could not be acquired (Figure 8a).

The “hot” strategy is identified as photothermal ablation of the potential metastatic LNs under the irradiation of 793 nm laser. We first verified the availability of this strategy by using 4T1‐luciferase cell line. After the photothermal therapy, the bioluminescent signal of lymph node metastasis also could not be detected (Figure 8a). A total of 16 tumor‐bearing mice (10 d after 4T1 cells injection) were further randomly divided into four groups: PBS group, PBS & laser group, IDSe‐IC2F NPs group, and IDSe‐IC2F NPs & laser group. 24 h after paracancerous injection, the mice received the photothermal ablation. We found that the temperature of IDSe‐IC2F NPs treated LNs rose above 55 °C within 2 min after the 793 nm laser irradiation (Figure 8b,c). However, the temperature of PBS‐treated LNs only mildly increased to around 38 °C. The treatment effect of photothermal ablation was assessed by measuring the size of axillary LN. The representative images of mice with LN metastasis model 7 d after photothermal ablation are presented in Figure 8d. Swollen axillary LNs in PBS group, PBS & laser group, and IDSe‐IC2F NPs group were observed obviously. Whereas in the IDSe‐IC2F NPs & laser group, no obviously swollen axillary LN was detected. On the tenth day, all the axillary LNs were harvested. We also resected four normal axillary LNs from the normal nude mice as the blank control (Figure 8e). The axillary LNs in PBS group, PBS & laser group, and IDSe‐IC2F NPs group were significantly bigger than those in blank control group and IDSe‐IC2F NPs & laser group (Figure 8e,f). The results suggested that IDSe‐IC2F NPs labeling plus laser irradiation could significantly suppress the regional LNs metastasis. H&E staining was studied after the axillary LNs were harvested (Figure S14, Supporting Information). LNs in PBS group, PBS & laser group, and IDSe‐IC2F NPs group were occupied by a large amount of tumor cells. In the IDSe‐IC2F NPs & laser group, there were necrosis tissues and very few residual tumor cells.

### 2.9 In Vitro and In Vivo Biosafety of IDSe‐IC2F NPs

Considering the NPs were commonly accumulated in the liver, we selected normal liver cell line (L‐02) to assess the cytotoxicity of IDSe‐IC2F NPs in vitro. No obvious cytotoxicity was detected in vitro (Figure S15, Supporting Information). To further assess the biological toxicity of NPs, we intravenously injected the NPs into mice and carried out hepatic/renal function analysis and H&E staining of important organs at 1 d and four weeks after injection. No significantly acute or chronic hepatic and renal damage was observed (Figures S16 and S17, Supporting Information). The H&E‐stained tissues revealed that no noticeable acute damage (Figure S16, Supporting Information) and chronic damage (Figure S17, Supporting Information) or inflammatory lesion existed.

## 3 Discussion

Appropriate lymphadenectomy significantly improves the prognosis after surgeries for patients with cancer. Currently, ICG was the most widely used fluorophore to label the LN during surgery. A recent randomized clinical trial (RCT) has proven that ICG‐based NIR‐I fluorescence imaging guided LN dissection could noticeably improve the number of dissected LN in radical gastrectomy.^[^ ^23^ ^]^ Those results encourage us to explore the application value of NIR‐II fluorescence imaging‐guided lymphadenectomy, because NIR‐II fluorescence imaging possesses higher spatial resolution, larger penetration depth, and higher SBR than NIR‐I fluorescence imaging. Here, we synthesized organic NIR‐II fluorescent IDSe‐IC2F molecules and encapsulated them with DSPE‐PEG_2000_ to form IDSe‐IC2F NPs, which could easily label the LNs. Compared to the ICG, IDSe‐IC2F NPs are brighter in the lymphatic fluid and infiltrate into blood vessels more slowly when used via local administration. To design proper NPs for lymph node tracing, their size is one of the most important factors. Unlike in the tumor, the blood vessels in normal tissues are well‐developed, and their walls are intact. Therefore, after a local administration, NPs with proper size could only enter into the lymphatic capillaries and travel rapidly to the LNs, without entering into the blood vessels. In addition, the size of NPs should simultaneously be large enough, ensuring them phagocytosed by macrophage and remain in the lymph nodes long enough for staining.^[^ ^24^ ^]^ According to the previous study, an optimal size of lymph node tracer should range between 50 and 200 nm,^[^ ^25^ ^]^ while the mean diameter of our IDSe‐IC2F NPs is ≈52 nm. If the tracer is a NP with small size or a small molecule such as ICG, it can both get through the blood vessel wall and travel in lymphatic system rapidly, which eventually leads to entering the blood circulation quickly.^[^ ^24^ ^]^

In addition, the normal organ injury during the surgery is a troublesome issue in clinical practice. Sometimes, only visualizing diseased organ may be insufficient, as diseased organs are usually close to or surrounded by normal organs. Previous clinical studies on the NIR fluorescence imaging‐guided surgery were mostly based on single‐channel imaging.^[^ ^9^, ^26^ ^]^ A multichannel fluorescence imaging‐guided surgery platform would significantly improve the success rate and safety of surgery. Although the fluorescence imaging in NIR‐I window has been widely applied in different kinds of surgeries, the limitations of conventional imaging in NIR‐I window are the high photon scattering and the nonignorable background signal of normal tissue.^[^ ^26^, ^27^ ^]^ For this reason, the images of a certain organ in NIR‐I region always contain too obvious background signals to generate multichannel imaging. However, the autofluorescence in NIR‐II region is negligible which could acquire the image without obviously noise signals. Therefore, NIR‐II region is more suitable for multichannel imaging. It is also significantly facilitating the process of picture merging in NIR‐II window. Although some previously reported NIR‐II fluorophores could visualize tumor lesions or the LNs and help surgeons to remove the targeted tissues,^[^ ^11^, ^15^, ^28^ ^]^ they did not simultaneously label the surrounding normal tissues. The iatrogenic healthy structure injury remains inevitable. In our research, we highlighted the concept of multichannel NIR‐II fluorescence imaging‐guided surgery. Combining the NIR‐II fluorescent NPs with different optical properties, we not only labeled the LNs but also visualized the surrounding normal structures, including blood vessels and ureters. As the excitation spectra or the emission spectra are different among the three kinds of NPs, the signals in different NIR‐II fluorescence channels were controllable. When the signals of surrounding normal tissues were turned off, we could rapidly and accurately find the location of LNs. After then, we further lit up the important normal tissues to protect them during surgery. Therefore, careful resection of LNs with preservation of the important organs could be easily achieved. Here, we want to emphasize the immense application potential of the combination of different NIR‐II fluorescent probes in imaging‐guided surgery.

According to the previous study, most of the biological tissues have autofluorescence spectrum in the NIR‐II window.^[^ ^29^ ^]^ If the signal intensity of exogenous NIR‐II fluoroprobe is weak, the autofluorescence will affect the bioimaging seriously. Luckily, in our study, the NIR‐II fluorescence intensity of IDSe‐IC2F NPs is much higher than the intensity of tissue autofluorescence, which helps us acquire NIR‐II fluorescence images with high SBR. Another possible solution to overcome the influence of tissue autofluorescence is using NIR‐II fluorescence lifetime imaging. For wide‐field macroscopic/microscopic luminescence lifetime imaging, the luminescent probes (e.g., rare‐earth doped NPs) are demanded to have a long luminescent lifetime (longer than microsecond or more) which can be resolved by the high‐speed camera.^[^ ^30^ ^]^ However, the fluorescence lifetime of IDSe‐IC2F NPs is very short (only 281 ps) and not suitable for the application scenario of imaging‐guided surgery, which is based on wide‐field macroscopic fluorescence imaging. Nevertheless, IDSe‐IC2F NPs can be used for NIR‐II fluorescence lifetime microscopic imaging, which is usually based on the “point‐excitation and point‐detection” imaging mode and time‐correlated single‐photon counting method.

Furthermore, we surprisingly found that under NIR‐II fluorescence imaging, LNs showed “ring” shape rather than solid round shape. We speculated this characteristic might be destroyed by metastatic cancer cells, as they may occupy the hollow position of LNs. Thus, it is worthy to explore whether this characteristic could help to determine the presence of LN metastasis in the future research.

When using NPs for bioimaging, the most concerned problem is their potentially high systematic toxicity. However, IDSe‐IC2F NPs infiltrated into blood vessels very slowly when used via local administration during surgery. Besides, the primary tumor and the sentinel/regional LNs are usually removed together, and it indicates that the residual NPs would be very few as most of them are removed along with the surgical samples. Local but not systemic administration of NIR‐II fluorescent NPs could be much safer.

## 4 Conclusion

In summary, a novel kind of NIR‐II fluorescent nanoprobe was synthesized to label and trace LNs. Together with other kinds of NIR‐II fluorescent NPs with different optical features, multichannel fluorescence imaging‐guided surgery was successfully conducted. Photothermal ablation of metastatic LNs is introduced as a good complement to surgery. This platform offered a novel perspective through which we can conduct a more precise surgery.

## 5 Experimental Section

##### Synthesis and Characterization of IDSe‐IC2F Nanoprobes

Please see the Supporting Information for the details.

##### Collection of Human Lymphatic Fluid

A 56‐year‐old thyroid cancer patient received radical thyroidectomy and suffered from lymphatic leak after operation. The drainage was collected after fully informed. The process was supervised by the Clinical Research Ethics Committee of Sir Run Run Shaw Hospital of Zhejiang University School of Medicine.

##### Cell Lines and Cell Culture

Mouse breast cancer cell line (4T1) was purchased from the Type Culture Collection of the Chinese Academy of Sciences (Shanghai, China). L‐02 normal liver cell line was kindly provided by Cang Yong's lab, Zhejiang University. 4T1 cell line and L‐02 were cultured in RPMI‐1640 (Gibco, Cat. No. C11975500BT) supplemented with 10% FBS (Cellmax, Cat. No. SA102.02) and maintained at 37 °C with 5% CO_2_.

##### Establishment of a Luciferase‐Transfected 4T1 Cell Line

The lentivirus (pHBLV‐Luc‐Puro, lot number: 45070327) was purchased from Hanbio. 4T1 cells were precultured at a density of 1 × 10^5^ cells in six‐well plate. pHBLV‐Luc‐Puro virus was added at a multiplicity of infection (MOI) of 20. 4T1 cancer cells were then cultured for 24 h and further selected with 2 µg mL^−1^ puromycin for 3 d.

##### Animals and LN Metastasis Model

Rats (200 g) and BALB/c nude mice (*n* = 16, 6 weeks of age) were purchased from Shanghai Laboratory Animal Center and housed in the Laboratory Animal Research Center of Sir Run Run Shaw Hospital, Zhejiang University School of Medicine. All the animal protocols were assessed and approved by the Animal Laboratory Ethics Committee of Zhejiang University. To establish the axillary LN metastasis model, 5 × 10^5^ 4T1 breast cancer cells were suspended in 20 µL PBS and further injected into the fore foot pad of each mouse (Figure S13, Supporting Information). The sizes of axillary LNs were inspected 2, 4, 6, 8, and 10 d postinjection. About at the tenth day postinjection, obvious swollen axillary LNs were observed.

##### In Vivo NIR‐II Fluorescence Imaging of Different Kinds of LNs

The single‐channel NIR‐II fluorescence imaging system was described in a previous study.^[^ ^15^ ^]^ By using this imaging system, the NIR‐II fluorescence imaging of different kinds of LNs was conducted. The incandescent lamp was used to provide the background of images. The nude mice and rats were anesthetized and imaged under the 793 nm laser. The IDSe‐IC2F NPs were injected into the rear paw of the nude mouse to detect the sciatic and popliteal lymph nodes. A laparotomy in rats was performed to acquire the images of different LNs in the abdominal cavity, including perigastric LN, retroperitoneal LN, pericolic LN, and mesenteric LN. The image of rat's popliteal LN was acquired after shaving. The LNs in rats were labeled by injecting IDSe‐IC2F NPs into the corresponding area (Figure S8, Supporting Information).

##### Multichannel NIR‐II Fluorescence Imaging and Imaging‐Guided Surgery

As shown in Figure S10 (Supporting Information), a 793 nm laser and a 623 nm LED were used to provide excitation for the irradiation area. The 793 nm laser beam was collimated and expended by a lens. A 2D electronic‐cooling InGaAs camera (Tekwin System, China) was used to capture the NIR‐II fluorescence images. The 623 nm LED with wide emission spectrum not only excited the TQ‐BPN NPs but also provided the visual background of surgical area. The size of images recorded by this camera was 640 pixels × 512 pixels. A 1100 or a 1500 nm long‐pass filter was placed in front of the camera. The animals were placed ≈40 cm away from the camera. All the surgical processes were recorded under the irradiation of incandescent lamp or 623 nm LED. For triple‐channel NIR‐II fluorescence imaging, the TQ‐BPN NPs, IDSe‐IC2F NPs, and PEG‐CSQDs were used sequentially.

##### In Vivo NIR‐II Fluorescence Imaging‐Guided Resection of LNs

For the potentially metastatic LNs in nude mice, the IDSe‐IC2F NPs were injected into the paracancerous tissues, and NIR‐II fluorescence imaging‐guided system was used to locate the LNs. A 0.5–1 cm incision was made upon the skin above the LNs, and the LNs were removed then. The whole process was under irradiation of the incandescent lamp.

For the abdominal LNs, the rats first received the laparotomy and imaged by using the nanoparticles. Then, the afore‐mentioned multichannel NIR‐II fluorescence imaging‐guided system was used to guide the lymphadenectomy.

##### In Vivo Photothermal Ablation of Metastatic LNs

When the obviously swollen LNs were observed, the mice were divided into four groups. In two groups, the mice were subcutaneously injected with 25 µL 1× PBS in the paracancerous tissues. In the rest two groups, the mice were subcutaneously injected with 25 µL IDSe‐IC2F NPs (1 mg mL^−1^) in the paracancerous tissues. 24 h after the injection, with the help of the NIR‐II fluorescence imaging system, the position of axillary LN could be precisely located in the IDSe‐IC2F NPs treated group. Then, a 0.5–0.8 cm incision was made to expose the LN. In the PBS‐treated groups, the incision was made according to the authors' experience. Upon the 793 nm laser irradiation (1.5 W cm^−2^), the temperature alterations of LN were measured by an infrared thermal camera. When the photothermal ablation was completed, the incision was sutured to protect the treatment region.

##### H&E Staining

All the LNs, including normal LNs and metastatic LNs, were fixed in 4% paraformaldehyde after harvested for 12 h. The LNs were further dehydrated, embedded in paraffin, and sectioned into 3 µm thick slides. H&E staining was then performed according to the manufacturer's protocol of H&E kit (Beyotime Institute of Biotechnology, Cat. No. C0105). H&E staining images of LNs were acquired by using the upright microscope (Nikon eclipse 80i).

##### Data Analysis

Quantitative analysis of fluorescence intensity was conducted by Image J software (Version 1.6.0, National Institutes of Health, USA). Origin Pro software (Version 9.0, OriginLab Company, USA) was used to generate the graphs. The SPSS 20.0 version was used to perform the statistical analysis. Paired two‐tailed Student's *t*‐test was conducted to assess the differences between different groups. A *P*‐value < 0.05 was considered as statistically significant. For the continuous variables, the data were presented as mean ± SD.

## Conflict of Interest

The authors declare no conflict of interest.

## Supporting information

Supporting Information

Supplemental Video 1

Supplemental Video 2

Supplemental Video 3

Supplemental Video 4

Supplemental Video 5

Supplemental Video 6

Supplemental Video 7

## Data Availability

The data that support the findings of this study are available from the corresponding author upon reasonable request.
