# Supplementary material for: Nanoprobes‐Assisted Multichannel NIR‐II Fluorescence Imaging‐Guided Resection and Photothermal Ablation of Lymph Nodes
Source: Adv Sci (Weinh). 2021 Feb 15;8(9):2003972. doi: 10.1002/advs.202003972 (PMC8097375; doi:10.1002/advs.202003972)
Supplement: Supplementary file 1 — Supporting Information [file ADVS-8-2003972-s005.pdf]

© 2021 Wiley-VCH GmbH

ADVANCED SCIENCE Open Access

Supporting Information

for *Adv. Sci.,* DOI: 10.1002/advs.202003972

Nanoprobes-assisted multi-channel NIR-II Fluorescence Imaging-guided Resection and Photothermal Ablation of Lymph Nodes

*Xiaoxiao Fan, Yirun Li, Zhe Feng, Guoqiao Chen, Jing Zhou, Mubin He, Lan Wu, Shengliang Li, * Jun Qian, * and Hui Lin**

Nanoprobes-assisted multi-channel NIR-II fluorescence imaging-guided resection and photothermal ablation of lymph nodes

Xiaoxiao Fan, Yirun Li, Zhe Feng, Guoqiao Chen, Jing Zhou, Mubin He, Lan Wu, Shengliang Li, Jun Qian,* Hui Lin**

Dr X. Fan, Y. Li, G. Chen, Prof. J. Qian, Prof. H. Lin
Department of General Surgery, Sir Run Run Shaw Hospital, School of Medicine, Zhejiang University, Hangzhou, 310000, PR China. E-mail: 369369@zju.edu.cn; qianjun@zju.edu.cn

Dr. X. Fan, Z. Feng, J. Zhou, M. He, Prof. L. Wu, Prof. J. Qian
State Key Laboratory of Modern Optical Instrumentations, Centre for Optical and Electromagnetic Research, College of Optical Science and Engineering, International Research Center for Advanced Photonics, Zhejiang University, Hangzhou 310058, PR China.
Dr. S. Li
College of Pharmaceutical Sciences, Soochow University, Suzhou 215123, PR China. E-mail: lishengliang@iccas.ac.cn
Dr. S. Li
Center of Super-Diamond and Advanced Films (COSDAF), Department of Chemistry, City University of Hong Kong, 83 Tat Chee Avenue, Kowloon, Hong Kong 999077, PR China.

Supporting Information

Methods

Molecule synthesis and characterization

IDSe-IC2F was synthetized *via* Knoevenagel condensation as our previous work [1]. Briefly, the compound 2-(5,6-difluoro-3-oxo-2,3-dihydro-1H-inden-1-ylidene)malono-nitrile (115 mg, 0.5 mmol) and 7,7'-(4,4,9,9-tetrakis(4-hexylphenyl)-4,9-dihydro-s-indaceno[1,2-b:5,6-b']bis(selenophene)-2,7-diyl)bis(2,3-dihydrothieno[3,4-b][1,4]dio- xine-5-carbaldehyde) (67 mg, 0.05 mmol) was mixed and dissolved with chloroform. After the pyridine was poured, the above mixture was treated with N_2 protection and then refluxed for 10 h reaction. To separate the chemical, the resultant was treated with a precipitation of methanol and then extracted with dichloromethane, with a further water washing. After these treatments, the crude outcome was further purified using the silica gel column chromatography to provide a black solid (100 mg, 56%). ^{1}H NMR (600 MHz, $CDCl_3$-*d*) δ 9.00 (s, 2H), 8.51-8.48 (dd, *J* = 9.9, 6.5 Hz, 2H), 7.74 (s, 2H), 7.62-7.59 (t, *J* = 7.5 Hz, 2H), 7.45 (s, 2H), 7.18-7.16 (d, *J* = 8.3 Hz, 8H), 7.12-7.10 (d, *J* = 8.3 Hz, 8H), 4.56-4.55 (dd, *J* = 5.0, 2.7 Hz, 4H), 4.47-4.46 (dd, *J* = 4.7, 2.8 Hz, 4H), 2.60-2.58 (m, 8H), 1.64-1.59 (m, 10H), 1.37-1.34 (m, 8H), 1.31-1.29 (m, 16H), 0.89-0.87 (t, *J* = 6.8 Hz, 12H). ^{13}C NMR (150 MHz, $CDCl_3$-*d*) δ 186.69, 159.50, 158.57, 154.36, 152.41, 151.83, 142.13, 141.02, 139.02, 138.80, 137.02, 135.32, 131.26, 128.75, 128.06, 125.61, 118.50, 118.34, 115.09, 114.83, 113.71, 112.36, 67.60, 66.32, 65.01, 64.31, 35.74, 31.88, 31.49, 29.28, 22.74, 14.25. HRMS (MALDI-TOF): m/z: $[M]^+$ calcd for $C_{102}H_{86}F_4N_4O_6S_2Se_2$, 1762.426; found, 1762.727.

Nanoprobes fabrication

To achieve good water-solubility, the IDSe-IC2F was co-assembled with amphipathic polymer DSPE-PEG_{2000} to form nanoparticles with PEG coating through typical nanoprecipitation method. The resulted IDSe-IC2F NPs were purified by a 0.22 μm filter to receive a uniform NPs solution, and then stored in 4 °C freezer for the

following usage.

TQ-BPN NPs and PEG-CSQDs were kindly provided by Ben Zhong Tang' lab and Mingxi Zhang' lab, respectively[2,3].

Measurement of absorption and fluorescence spectra

The absorption spectra of the three kinds of nanoparticles (NPs) in water were measured by using a Shimadzu UV-2550 UV-vis-NIR scanning spectrophotometer. The fluorescence emission spectra of NPs in water were collected by a lab-built system based on a PG2000 spectrometer (Ideaoptics Instruments) and a 2000C spectrometer (Everuping Optics Corporation).

Size distribution and zeta potential of IDSe-IC2F NPs

The morphology of IDSe-IC2F NPs was observed by using transmission electron microscope (TEM, JEOL JEM-1220, 160 kV under the bright field mode). Dynamic light scattering (DLS) analysis of IDSe-IC2F NPs and zeta potential were conducted on a Malvern Zetasizer Nano ZS size analyzer at room temperature.

Measurement of photothermal conversion efficiency

The aqueous dispersion of IDSe-IC2F NPs (150 μl, 0.1 mg/ml) was irradiated by a 793 nm laser (0.72 W/cm^2). The laser was switched off when the temperature of dispersion reached the maximum, and the changes of temperature were recorded every ten seconds until it cooled down to the room temperature. The photothermal conversion efficiency (η) was calculated according to the following equation:

$$\eta = \frac{hS(T_{max} - T_{surr}) - Q_{dis}}{I(1 - 10^{-A_{793}})}$$

The h represents the heat transfer coefficient, S represents the container's surface area, T_{max} represents the maximum temperature, T_{surr} represents the room temperature, Q_{dis} represents the heat dissipation caused by the light absorption of container, I represents the power of laser (0.36 W), and A_{793} is the absorbance of NPs in water (0.1 mg/ml) at 793 nm (OD = 1.93).

The value of hS can be calculated according to the equation:

$$hS = \frac{m_D C_D}{\tau_s}$$

Where m_D is the mass of aqueous dispersion of IDSe-IC2F NPs (0.15 g), C_D is the

heat capacity of solvent (water, 4.2 J/g), and τ_s is the time constant.

Photostability test

The photostability of IDSe-IC2F NPs (concentration 0.5 mg/ml) was explored under the continuous irradiation of 793 nm laser (1.5 W/cm^2) for 60min. The NIR-II fluorescence image was taken every 10 minutes.

Photothermal stability test

The photothermal stability of IDSe-IC2F NPs was assessed by repeated laser irradiation. 300 μl aqueous dispersion of NPs (0.2 mg/ml) in 1.5 ml EP tube was irradiated by the 793 nm laser with power density of 0.6 W/cm^2. When the temperature raised to the maximum, the laser was turned off, and the aqueous dispersion cooled to the room temperature. The temperature changes were recorded every 10 seconds during this period. A total of five cycles were monitored.

***In vitro* cytotoxicity analysis**

L-02 cells (5×10^3 per well) were pre-cultured in 96 well plates overnight, and the medium was then replaced by 200 μl fresh DMEM medium containing IDSe-IC2F NPs with different concentrations (0, 2, 5, 10, 20, and 50 μg/ml). The treated cells were further incubated for 48 hours. Subsequently, the medium was replaced by 200 μl fresh medium, and 20 μL MTT with the concentration of 5 mg/ml was then added. After incubated for 3 hours, optical densities (OD) at 490 nm and 570 nm were measured by an enzyme-linked immune sorbent assay (ELISA) reader.

***In vivo* biological toxicity analysis**

Twelve mice were randomly divided into two groups. The mice in control group were injected with 200 μl DI water, and the mice in NPs group were injected with 200 μl NPs (1 mg/ml). Six mice, three from the control group and three from the NPs group, were sacrificed one day after injection to assess the acute biological toxicity. The rest mice were sacrificed four weeks after injection to assess the chronic biological toxicity. The main organs were fixed in 4% paraformaldehyde for 24 hours at room temperature and then embedded in paraffin, sectioned, and stained with hematoxylin and eosin.

"(NPs@H2O: ~ 52 nm" is top text? No.

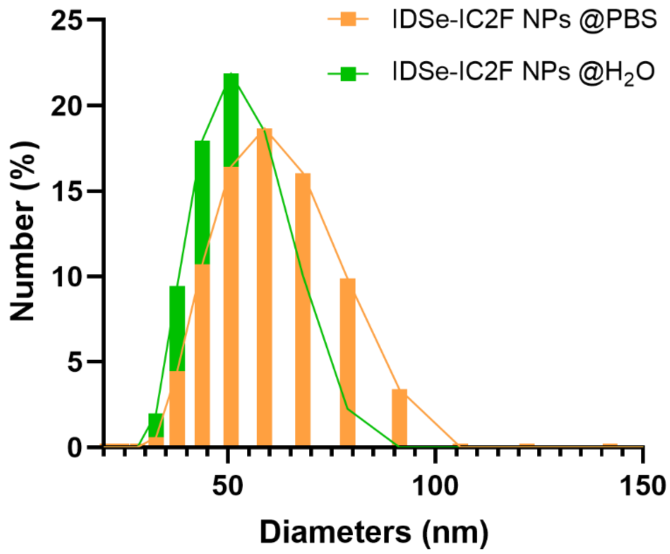

Supplementary Figure 1. The mean diameters of IDSe-IC2F NPs in different media ($NPs@H_2O$: ~ 52 nm; NPs@PBS: ~ 58 nm).

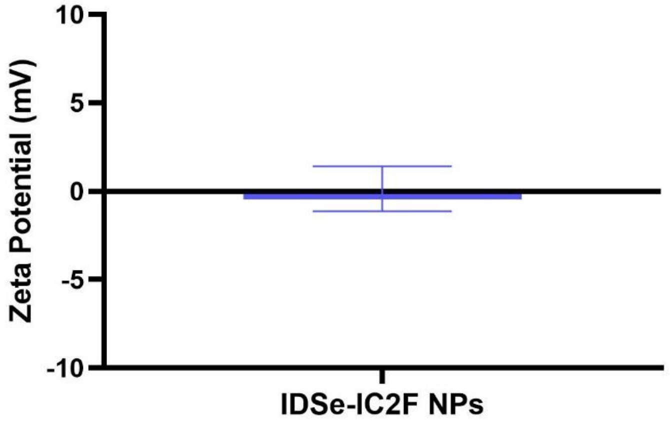

Supplementary Figure 2. Zeta potential of IDSe-IC2F NPs in water (n=3, Mean ± SD).

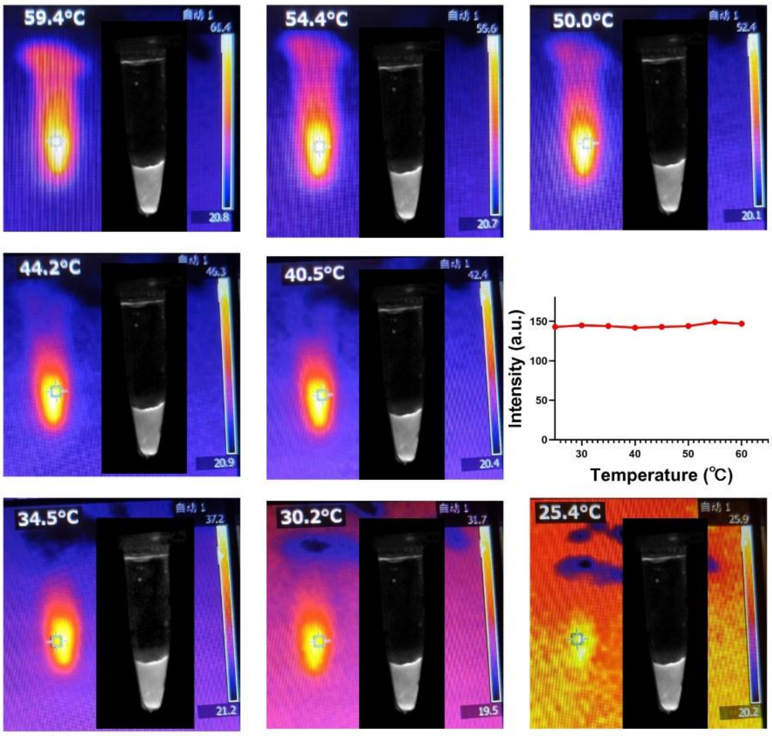

Supplementary Figure 3. The NIR-II fluorescence imaging of IDSe-IC2F NPs in EP tube at different temperatures. No obvious alteration of fluorescence intensity could be observed.

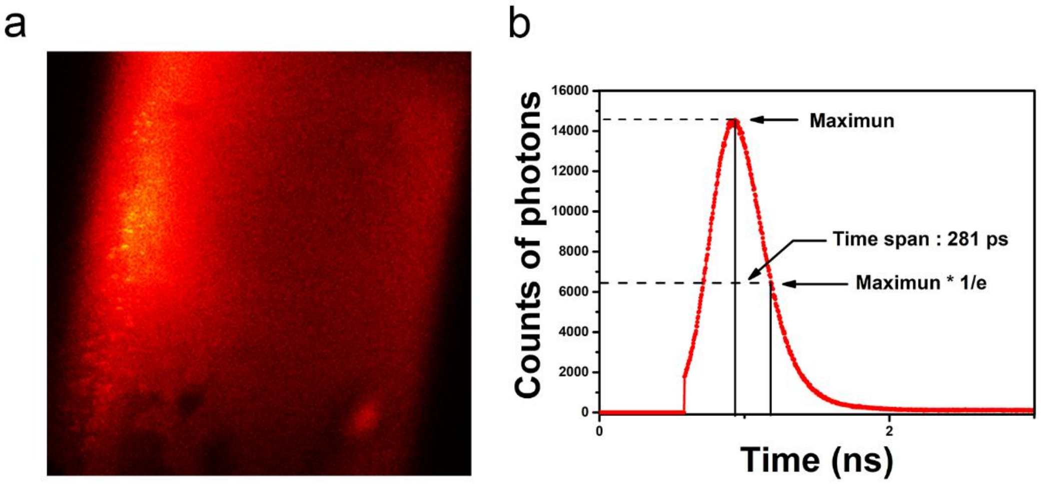

Supplementary Figure 4. (a) The NIR-II fluorescence lifetime image of IDSe-IC2F NPs in capillary tube; (b) Fluorescence decay curve measured based on (a), showing the NIR-II fluorescence lifetime of IDSe-IC2F NPs is 281 ps.

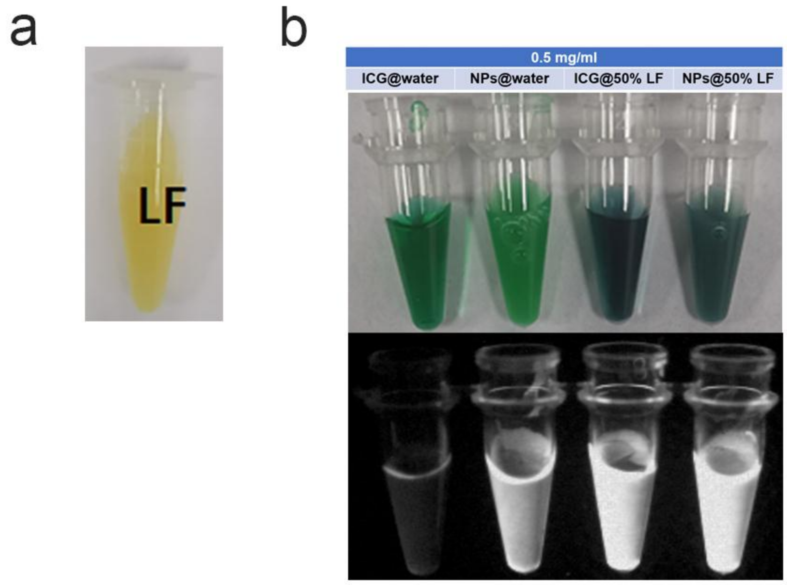

Supplementary Figure 5. (a) The picture of LF; (b) The bright-field and NIR-II fluorescence images of IDSe-IC2F NPs (0.5 mg/ml) and ICG (0.5 mg/ml) in water and in lymphatic fluid (LF) (optical filter: 1000 nm LP, power density: 7.5 mW/cm^2).

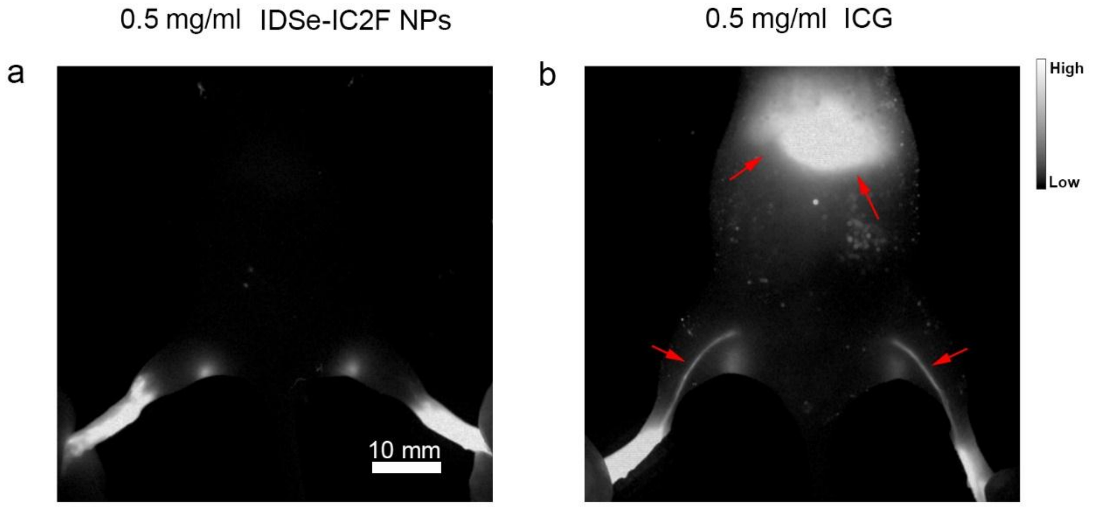

Supplementary Figure 6. The difference of NIR-II fluorescence images of nude mice, whose rear paw was injected with different fluorescent probes: aqueous dispersion of (a) IDSe-IC2F NPs (43 mW/cm^2, 100 ms) and (b) ICG (43 mW/cm^2,

100 ms).

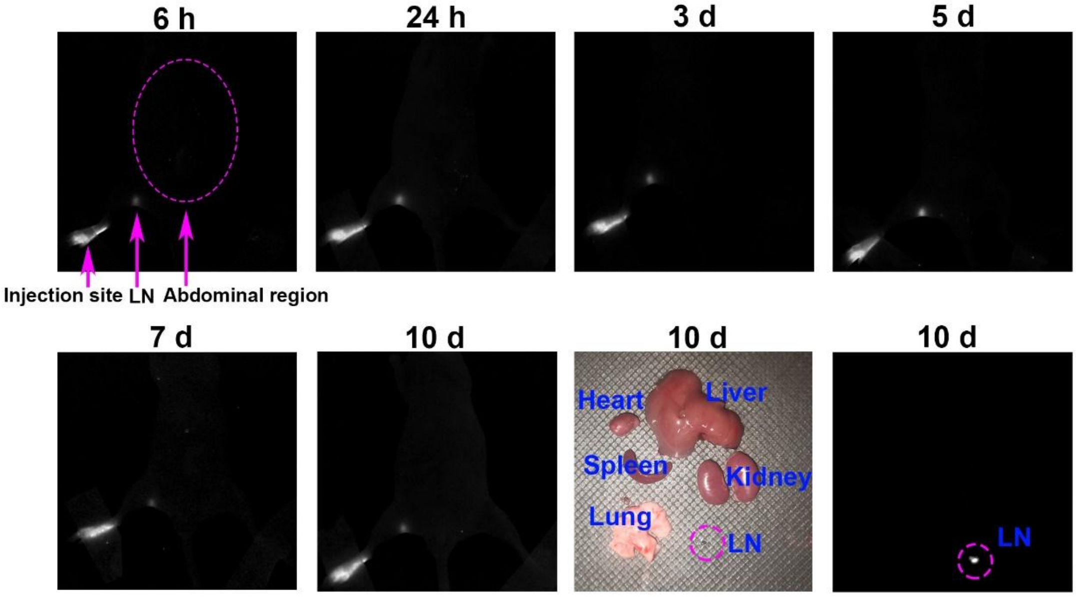

Supplementary Figure 7. *In vivo* NIR-II fluorescence imaging of mice at 6 hours, 1, 3, 5, 7 and 10 days after receiving a local administration of IDSe-IC2F NPs. *Ex vivo* bright-field and NIR-II fluorescence imaging of different organs of the treated mice at 10 days after injection.

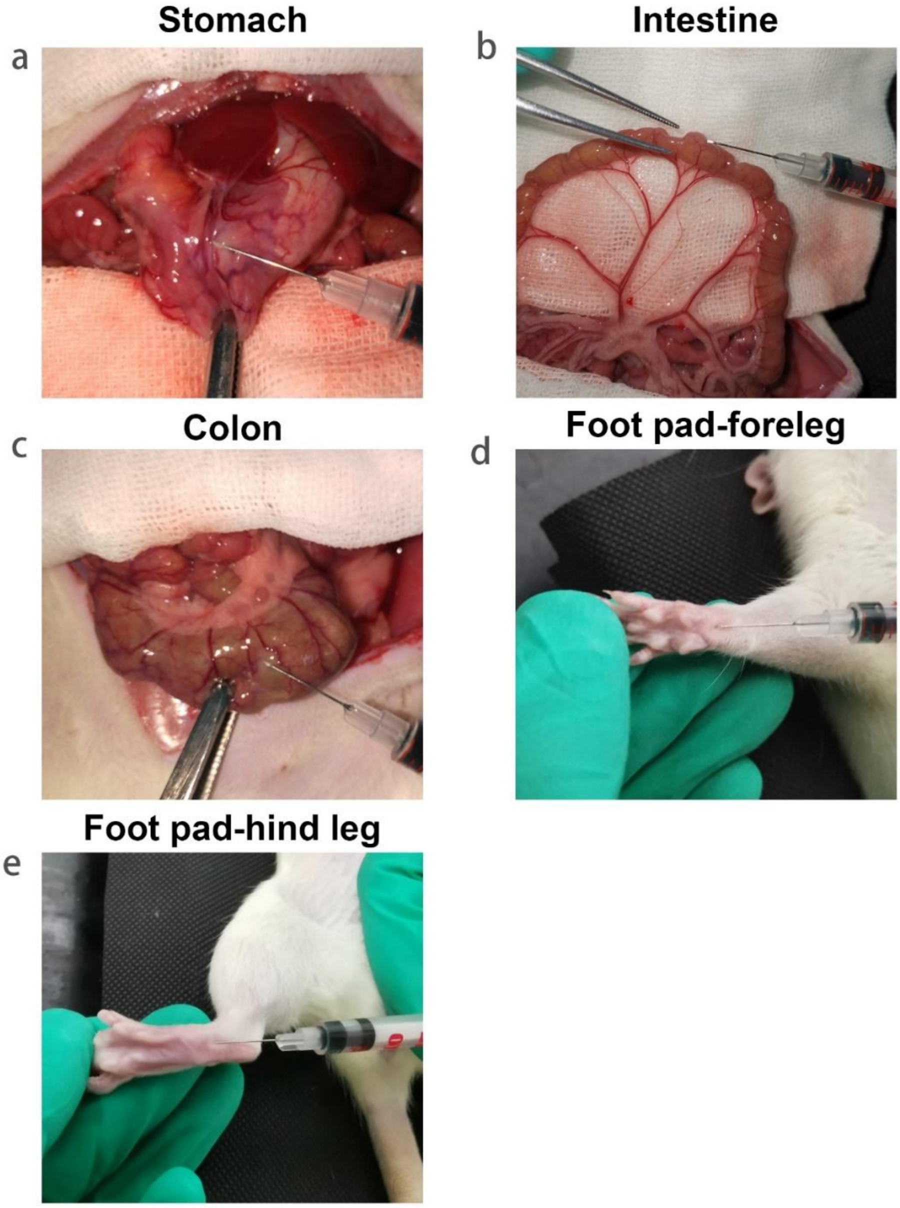

Supplementary Figure 8. Different injection sites of IDSe-IC2F NPs for labeling different kinds of LNs. (a) Perigastric LN; (b) Mesenteric LN; (c) Pericolic LN; (d) Axillary LN; (e) Popliteal and Retroperitoneal LN.

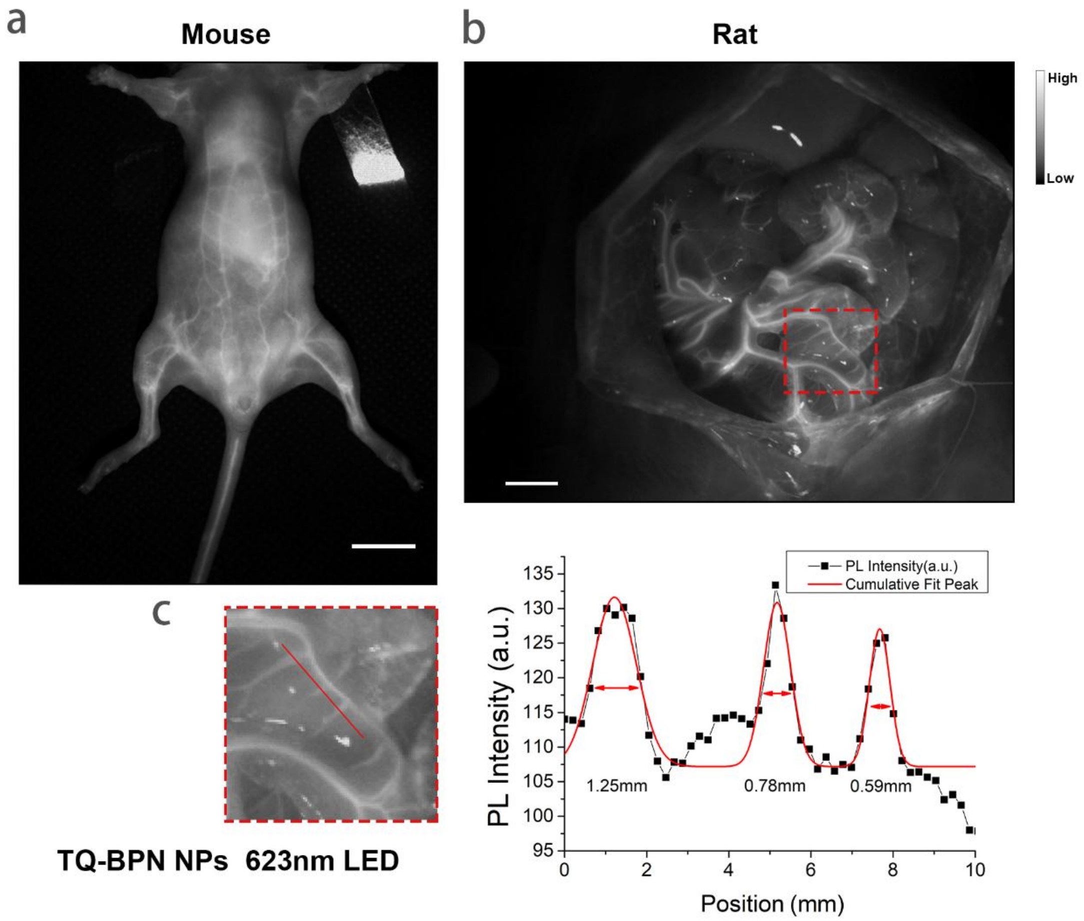

Supplementary Figure 9. *In vivo* NIR-II fluorescence whole-body imaging of a nude mouse and abdominal vascular imaging of a rat based on TQ-BPN NPs. (A) Whole-body imaging of a mouse treated with TQ-BPN NPs upon the excitation of 623 nm LED. Scale bar: 10 mm. (B) Imaging of different abdominal vessels of the rat under the excitation of 623 nm LED. Scale bar: 10 mm. (C) The local magnified image and the cross-sectional fluorescence intensity profile along the red line shown in (C) a Gaussian function fitted to the profile (with FWHM).

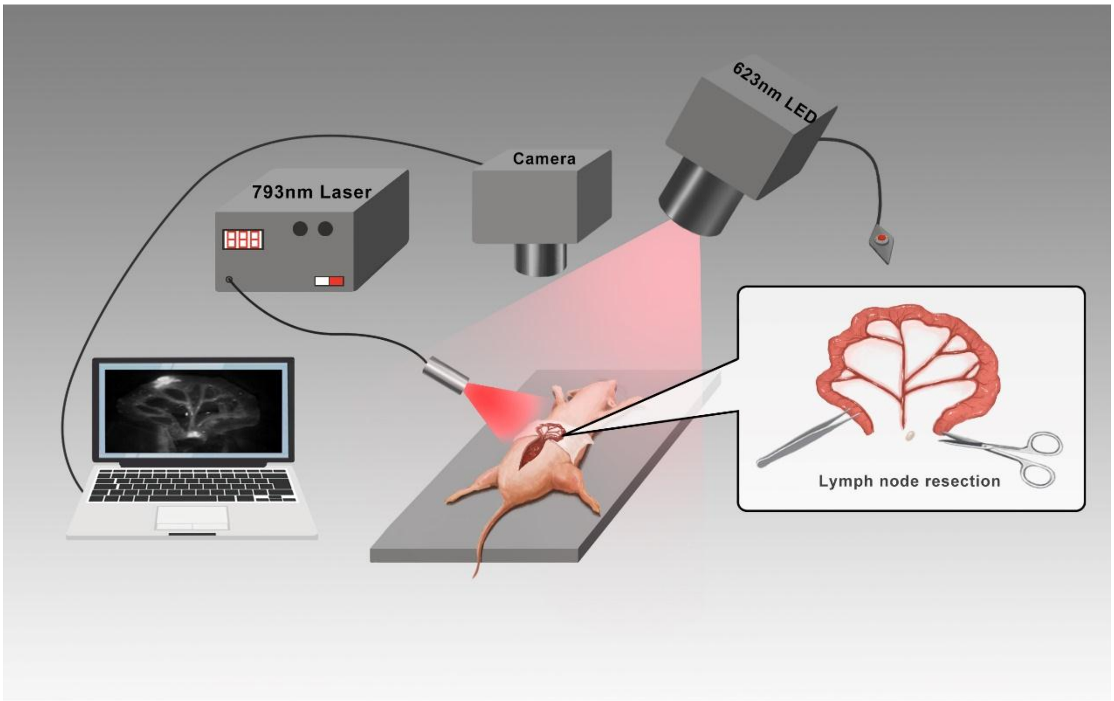

Supplementary Figure 10. Schematic illustration for dual-channel NIR-II fluorescence imaging-guided surgery system.

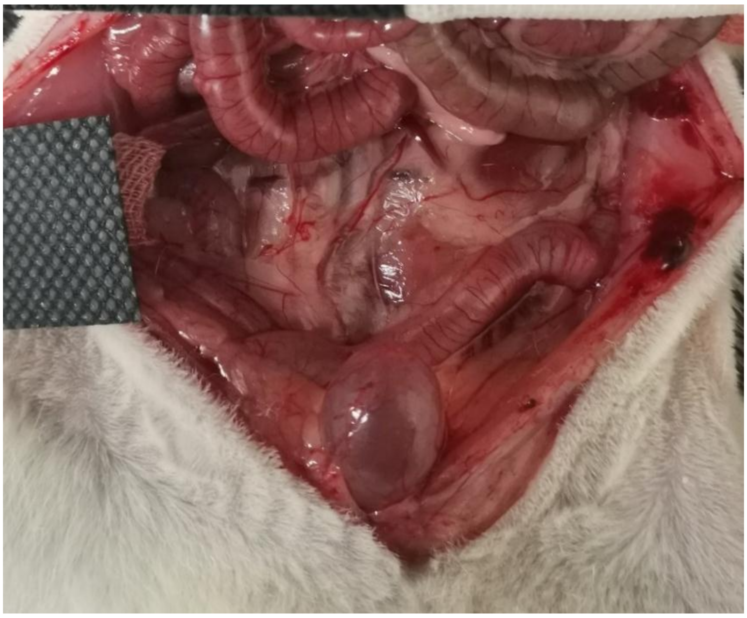

Supplementary Figure 11. The photograph of posterior peritoneum area in rat.

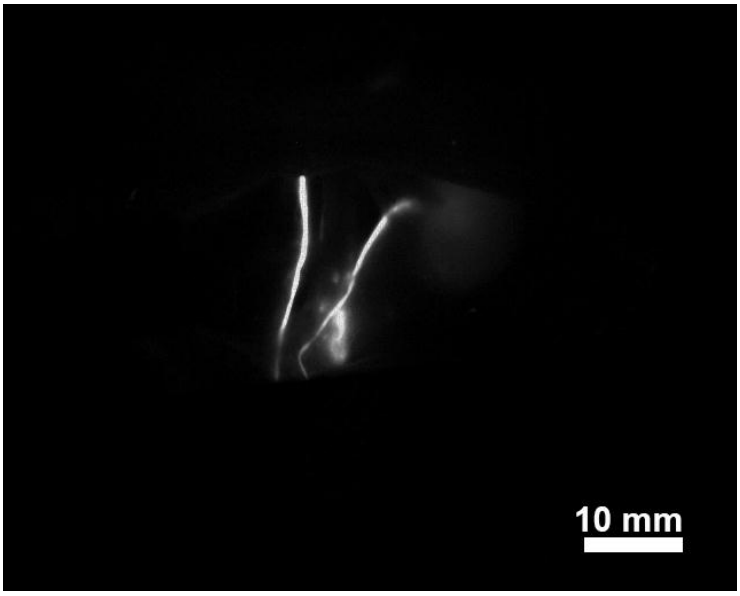

Supplementary Figure 12. A NIR-II fluorescence image of retroperitoneal area after retrograde ureteropyelography with PEG-CSQDs and LN labeling with IDSe-IC2F NPs, under the excitation of 793 nm laser (43 mW/cm^2), scale bar: 10 mm.

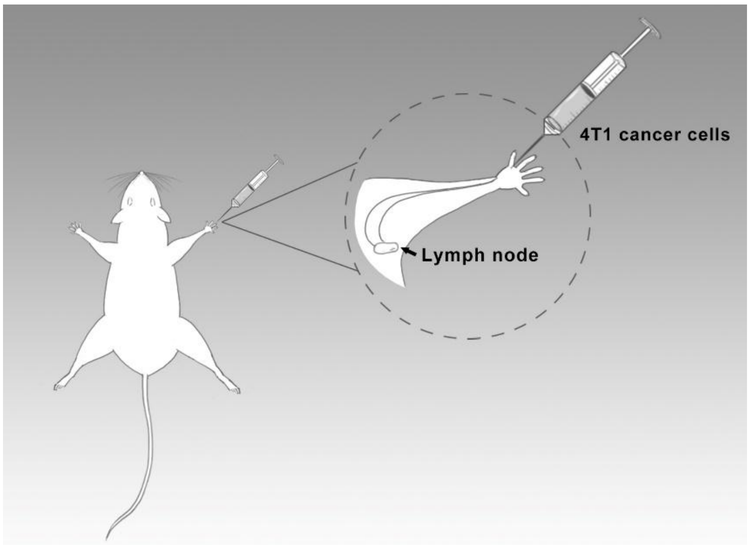

Supplementary Figure 13. Schematic illustration for establishing the model of axillary LN metastasis.

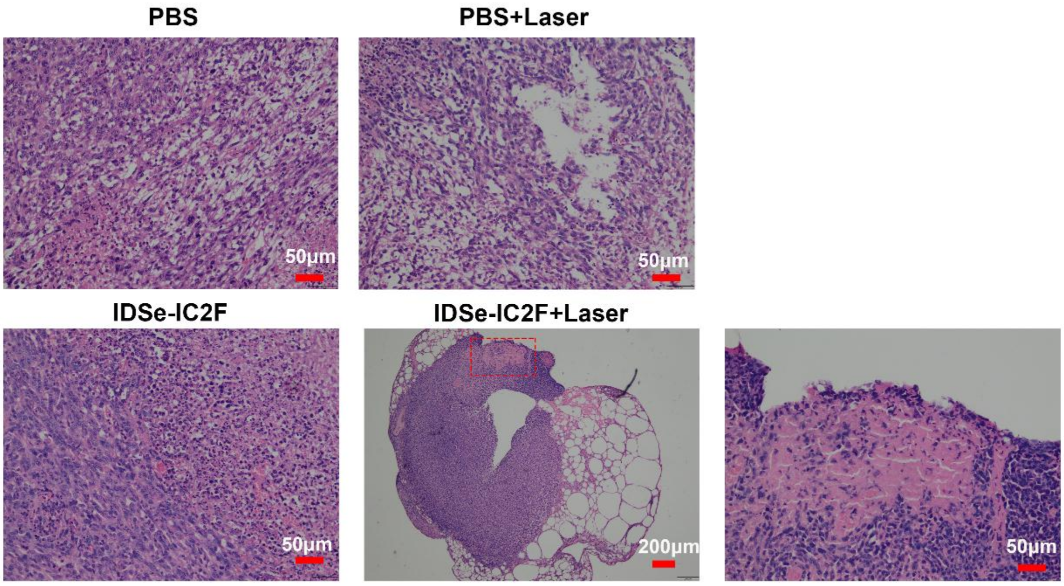

Supplementary Figure 14. H&E staining microscopic images of harvested LNs from four groups of mice, which were with LN metastasis model and received different treatments. (a) PBS groups. (b) PBS & laser group. (c) IDSe-IC2F NPs group. (d) and (e) IDSe-IC2F NPs & laser group.

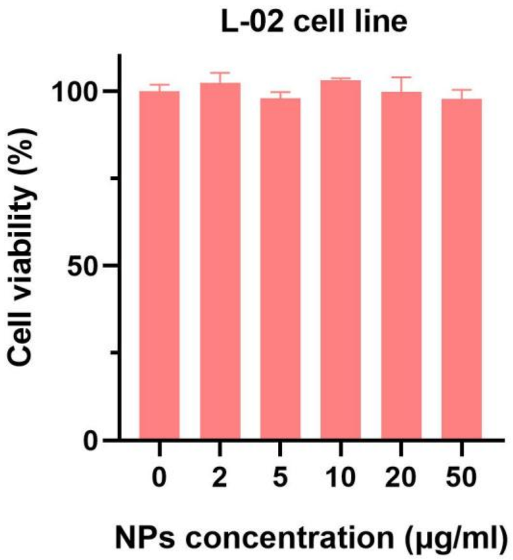

Supplementary Figure 15. Cell viability of L-02 cells after incubated with RPMI-1640 (10% FBS) containing IDSe-IC2F NPs with various concentrations for 48 h.

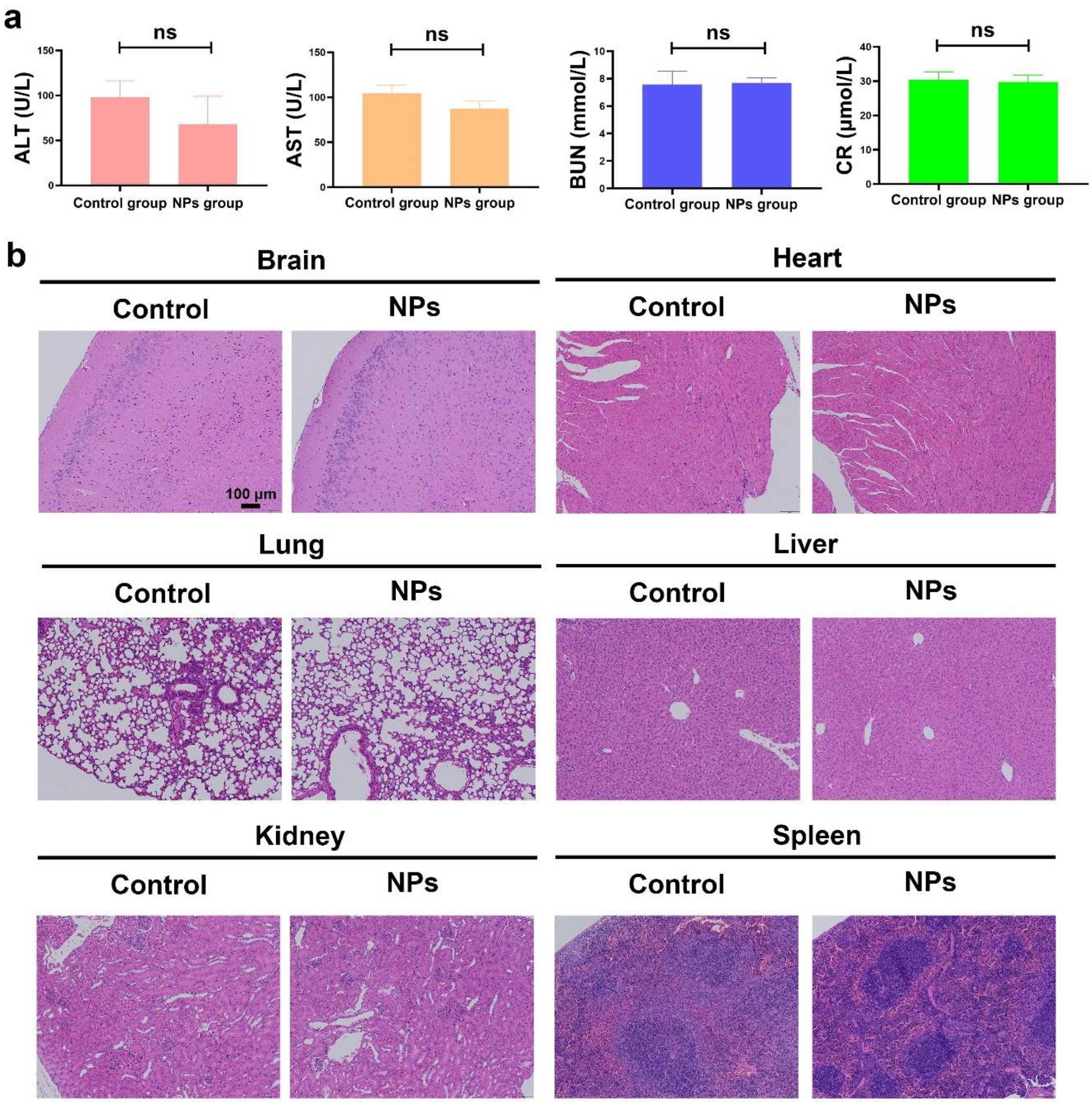

Supplementary Figure 16. Acute biological toxicity analysis, a) The hepatic and renal functions test of mice, which have been treated for 24 h. NPs group represents the mice treated with IDSe-IC2F NPs (200 μl, 1 mg/ml, n = 3); Control group represents the mice treated with DI water (200 μl, n = 3). b) H&E staining of tissue sections of different organs from the mice in two groups (n = 3).

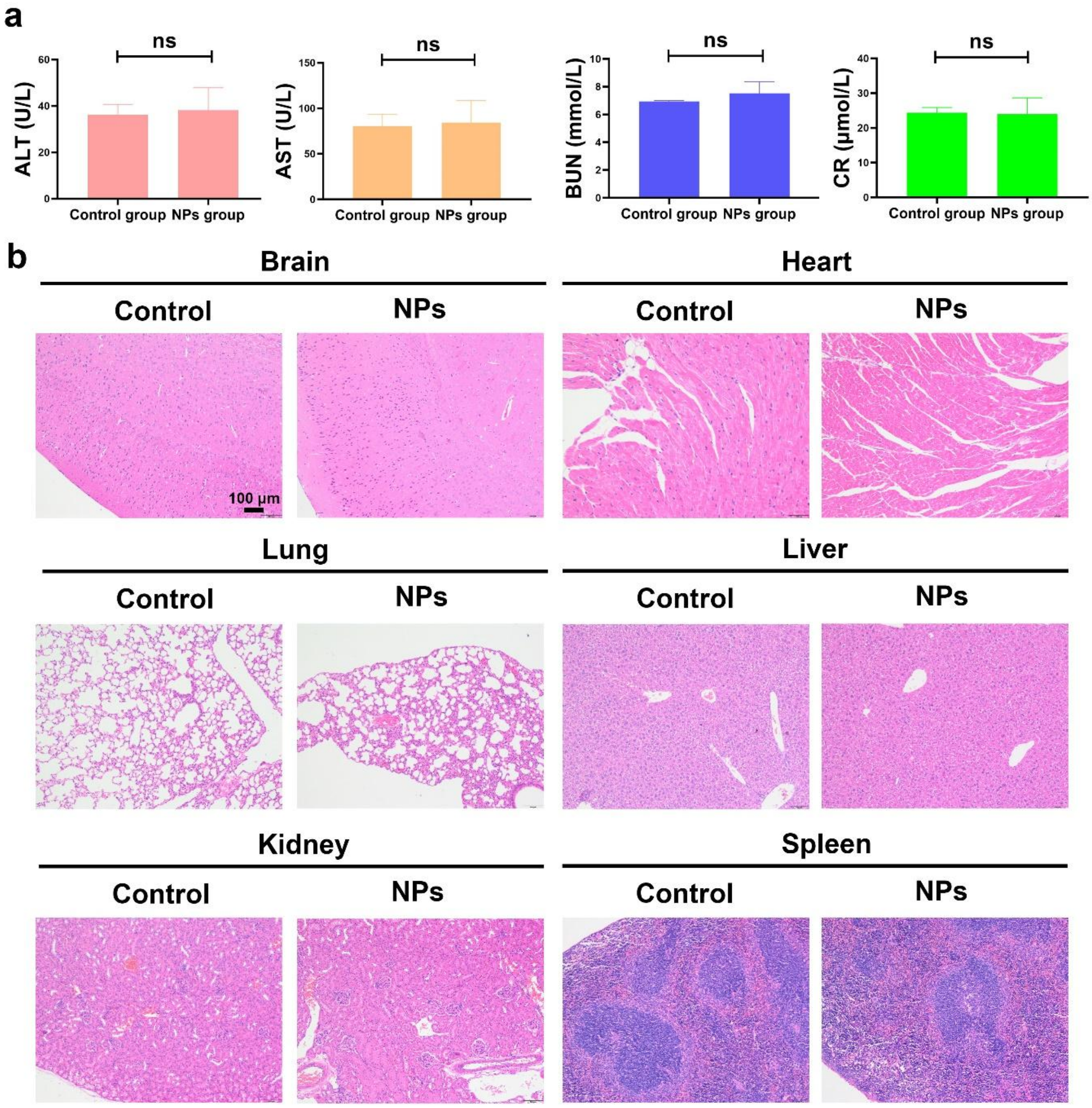

Supplementary Figure 17. Chronic biological toxicity analysis, a) The hepatic and renal functions test of mice, which have been treated for four weeks. NPs group represents the mice treated with IDSe-IC2F NPs (200 μl, 1 mg/ml, n = 3); Control group represents the mice treated with DI water (200 μl, n = 3). b) H&E staining of different organs from the mice in two groups (n = 3).

Supplementary video 1. The process of the lymphatic drainage from the follicles to the LNs.

Supplementary video 2. Dual-channel NIR-II fluorescence imaging-guided lymphadenectomy for mesenteric LN to avoid the injury of major blood vessels.

Supplementary video 3. Dual-channel NIR-II fluorescence imaging-guided lymphadenectomy for gastric LN to avoid the injury of major blood vessels.

Supplementary video 4. Single-channel NIR-II fluorescence imaging-guided lymphadenectomy for gastric LN.

Supplementary video 5. Dual-channel NIR-II fluorescence imaging-guided lymphadenectomy for retroperitoneal LN to avoid the injury of ureters.

Supplementary video 6. Simulating iatrogenic ureteral injury during the dual-channel NIR-II fluorescence imaging-guided lymphadenectomy.

Supplementary video 7. NIR-II fluorescence imaging-guided lymphadenectomy for LN metastasis.

Reference:

1 Li S. *et al.* Molecular Programming of Bright Semiconducting Oligomers for In Vivo Deep-Brain Microscopy in NIR-II Biowindow. *Submitted*.

2 Qi, J. *et al.* Real-Time and High-Resolution Bioimaging with Bright Aggregation-Induced Emission Dots in Short-Wave Infrared Region. *Adv Mater* **30**, e1706856, doi:10.1002/adma.201706856 (2018).

3 Zhang, M. *et al.* Bright quantum dots emitting at approximately 1,600 nm in the NIR-IIb window for deep tissue fluorescence imaging. *Proc Natl Acad Sci U S A* **115**, 6590-6595, doi:10.1073/pnas.1806153115 (2018).
